# A worldwide multicentre evaluation of the influence of deterioration or improvement of acute kidney injury on clinical outcome in critically ill patients with and without sepsis at ICU admission: results from The Intensive Care Over Nations audit

**DOI:** 10.1186/s13054-018-2112-z

**Published:** 2018-08-03

**Authors:** Esther Peters, Massimo Antonelli, Xavier Wittebole, Rahul Nanchal, Bruno François, Yasser Sakr, Jean-Louis Vincent, Peter Pickkers

**Affiliations:** 1 Department of Intensive Care Medicine, Radboud University Medical Center, HP:710, PO Box 9101, 6500 HB Nijmegen, The Netherlands; 2 Department of Intensive Care and Anesthesiology, Università Cattolica Del Sacro Cuore, Largo A. Gemelli 8, 00168 Rome, Italy; 3 Critical Care Department, Cliniques universitaires St Luc, UCL, Avenue Hippocrate 10, 1200 Brussels, Belgium; 4 Department of Medicine, Medical College of Wisconsin, 8701 Watertown Plank Rd, Milwaukee, WI 53226 USA; 5 Service de Réanimation Polyvalente, CHU Dupuytren, 2, avenue Martin Luther King, 87042 Limoges cedex, France; 6 Department of Anesthesiology and Intensive Care, Uniklinikum Jena, Am Klinikum 1, 07747 Jena, Germany; 7 Department of Intensive Care, Erasme University Hospital, Université Libre de Bruxelles, route de Lennik 808, 1070 Brussels, Belgium

**Keywords:** Critical care outcomes, Epidemiology, Renal replacement therapy

## Abstract

**Background:**

Acute kidney injury (AKI) is a common complication of critical illness and is associated with worse outcomes. However, the influence of deterioration or improvement in renal function on clinical outcomes is unclear. Using a large international database, we evaluated the prevalence and evolution of AKI over a 7-day period and its effects on clinical outcomes in septic and non-septic critically ill patients worldwide.

**Methods:**

From the 10,069 adult intensive care unit (ICU) patients in the Intensive Care Over Nations database, all those with creatinine and urine output data were included in this substudy. Patients who developed sepsis during the ICU stay (≥ 2 days after admission) were excluded. AKI was evaluated within 72 hours after admission and before discharge/death up to day 7 according to the Acute Kidney Injury Network (AKIN) criteria.

**Results:**

A total of 7970 patients were included, 59% of whom met AKIN criteria for AKI within the first 72 hours of the ICU stay. Twenty-four per cent of patients had sepsis on admission, of whom 68% had AKI, compared to 57% of those without sepsis on admission (*p* < 0.001). AKIN stage 3 (40% vs 24%, *p* < 0.001) and use of renal replacement therapy (20% vs 5%, *p* < 0.0001) were more prevalent in patients with sepsis. Patients with sepsis and AKIN stage 3 were less likely to improve to a lower stage during the 7-day follow-up period than non-septic patients with AKIN stage 3 (21% vs 32%, *p* < 0.0001). In-hospital mortality was related to severity of AKI and was reduced in patients in whom AKI improved compared to those who remained stable or deteriorated, but remained higher than in patients without AKI, even if there was apparent full recovery at day 7.

**Conclusion:**

These findings illustrate the different kinetics of AKI in septic and non-septic ICU patients and emphasize the important impact of AKI on mortality rates even when there is apparent full renal recovery at day 7*.*

**Electronic supplementary material:**

The online version of this article (10.1186/s13054-018-2112-z) contains supplementary material, which is available to authorized users.

## Background

Acute kidney injury (AKI) is a serious complication in critically ill patients and is independently associated with poorer outcomes, creating a large burden on both patients and society [1, 2]. Approximately one out of five non-severe sepsis patients develop AKI [3], increasing to one to two-thirds of critically ill patients, and its prevalence is increasing [4–9]. Approximately 50% of ICU patients with AKI die and those patients surviving an episode of AKI have an increased risk of developing chronic kidney disease [10, 11]. Currently, there are no pharmacological therapeutic options available to prevent or treat AKI and management is limited to mitigating secondary haemodynamic and toxic renal insults and providing supportive measures, such as diuretics and renal replacement therapy (RRT). AKI can be caused by a variety of factors [10], but sepsis is the most important aetiology in the critically ill [12]. Sepsis-associated AKI is distinct from non-sepsis AKI, with differences in pathogenesis, patient characteristics and clinical outcomes [12–14].

Global epidemiological data comparing the clinical course and outcomes in septic and non-septic AKI are sparse. Further, while the impact of development of de-novo AKI on short and long-term outcomes is well recognized, the influence of deterioration or improvement in renal function on clinical outcomes after development of AKI remains unclear, although these aspects are of importance for clinical decision-making and future research.

The objective of this study, therefore, was to provide an overview of the prevalence and evolution of AKI and its effect on clinical outcomes in critically ill adult patients worldwide to provide greater insight into the global burden of this condition. Using a large global registry of critically ill patient data collected during the Intensive Care Over Nations (ICON) audit [15], we evaluated the effects of deterioration or improvement in AKI severity on outcomes from septic and non-septic AKI. We hypothesized that septic AKI would be associated with worse outcomes than non-septic AKI.

## Methods

### Study population

This was a substudy of the Intensive Care Over Nations (ICON) audit, a multicentre, worldwide audit conducted between 8 and 18 May 2012 to collect data on characteristics, including organ dysfunction, infection and outcomes, of adult critically ill patients worldwide. Full details of the methodology have been published previously [15] and are provided in Additional file 1. Briefly, participating centres were recruited by open invitation, through national scientific societies, international meetings and/or individual contacts. A list of participating centres is provided in Additional file 2. Participation was voluntary with no financial reimbursement. In each institution, the study was approved by the institutional research ethics committee in accordance with local ethical regulations. Informed consent was not required due to the observational and anonymous nature of the data collection. Participating centres prospectively collected data from all adult patients (> 16 years) admitted to the ICU during the study period, except those admitted for less than 24 hours for routine postoperative surveillance. Readmissions of previously included patients were not considered. For the purposes of this substudy, only patients with creatinine and urine output data were included. Patients who developed sepsis during the ICU stay (≥ 2 days after admission) were excluded because we wanted to evaluate the impact of sepsis on ICU admission on AKI outcomes.

### Definitions

Sepsis was defined as the occurrence of at least one failing organ (Sepsis-related Organ Failure Assessment (SOFA) score > 2 for respective organ system) combined with the presence of infection, as defined according to the International Sepsis Forum [16]. The presence of AKI was evaluated using the Acute Kidney Injury Network (AKIN) criteria [17]. AKI stages were determined according to the AKIN criteria, using the largest increase between two values of serum creatinine obtained maximally 48 hours apart within a maximum period of 72 hours after ICU admission or the urine output for the 24-hour period after ICU admission*.* As urine output was only recorded per 24-hour period in ICON, we adjusted the stage 1 and 2 urine output criteria (Additional file 3: Table S1). The criterion (urine output or serum creatinine) that led to the worst possible AKIN classification was used in each case. We chose to use the AKIN criteria rather than the more recent KDIGO criteria, because in the KDIGO definition of AKI the creatinine criterion relies on the percentage change over a period of 7 days (rather than 48 hours for the AKIN criteria) and our aim was to demonstrate the effect of deteriorating or resolving AKI during the first 7 days after ICU admission on outcome. To assess AKI deterioration or improvement, the last serum creatinine or urine output data available before discharge or death up to day 7 were used to define the AKIN stage, again using the criterion (urine output or serum creatinine) that led to the worst possible AKIN classification in each case. Patients who died were not penalized to a maximum stage. This AKIN stage was compared to the initial stage, and patients were identified as having deteriorated, improved or remained the same. Complete recovery was defined as improvement from AKIN stage 1, 2 or 3 to the No AKI category. The inotropic score was calculated to express the use of vasoactive/vasopressor agents, as described elsewhere [18].

Patients with comorbid chronic renal failure (CRF), indicated on the original ICON case report form by the ICON investigator, were analysed as a separate subgroup.

### Statistical analysis

All statistical analyses were conducted in the Department of Intensive Care of Erasme Hospital. For the purpose of this study, the world was divided into nine geographical regions, and individual countries were classified into three income groups in accordance with the gross national income (GNI) per person [14]. Data are expressed as mean and standard deviation (SD), median with interquartile range (IQR, first–third quartiles) or numbers and percentages. For continuous variables, normality assumption checking was performed by inspection of residual and normal plots and by using the Kolmogorov–Smirnov test. Difference testing between groups was performed using the generalized linear models procedure, Kruskal–Wallis test, Student’s *t* test, Mann–Whitney test, χ^2^ test or Fisher’s exact test, as appropriate.

To describe the prevalence of patients receiving RRT up to day 7, data were imputed by the last observation carried forward in the case of discharge or death. If a patient died before day 60, the ICU and hospital length of stay (LOS) was each set to 60 days.

Data were censored at day 60. In addition, discharge of a patient was considered a competing risk factor for the occurrence of death. A competing risk regression model [19] was used to estimate crude and adjusted hazard ratios (HRs) and their 95% CIs for ICU and hospital mortality according to the AKI stage or CRF group. To determine the adjusted relative risk of in-ICU or in-hospital death, we developed a multivariable competing risk proportional hazard regression model, stratified according to the presence or not of sepsis. Other confounding variables considered included age, sex, Acute Physiology and Chronic Health Evaluation (APACHE) II score without age and renal components, type of admission, source of admission, reason for admission, the need for mechanical ventilation or RRT on admission to the ICU, comorbidities and the inotrope score. We also adjusted for ICU and hospital-related organizational factors including type of hospital, ICU specialty, total number of ICU patients in 2011 and number of staffed ICU beds. Geographic region and GNI were also considered. Collinearity between variables was excluded before modelling and the time-dependent covariate method was used to check the proportional hazard assumption of the model. The cumulative incidence functions of death according to AKIN stage within the first 72 hours of admission or CRF were plotted and Gray’s test was used to test cause-specific death differences [20, 21]. To quantify the association between the direction of the evolution of the AKI and sepsis, a cumulative link mixed-effects ordinal response model with logit link function was fitted. The random intercept model was used. The longitudinal ordinal response variable, AKIN stage, was the main dependent. The explanatory variables were the time points of AKIN stage assessment (admission/follow-up), sepsis (yes/no) and their interaction [22, 23]. Two-sided *p* < 0.05 was considered statistically significant. Data were analysed using IBM® SPSS® Statistics software, version 23 for Windows (IBM, Armonk, NY, USA) and R software, version 2.10.1 (CRAN project).

## Results

**Fig. 1:**
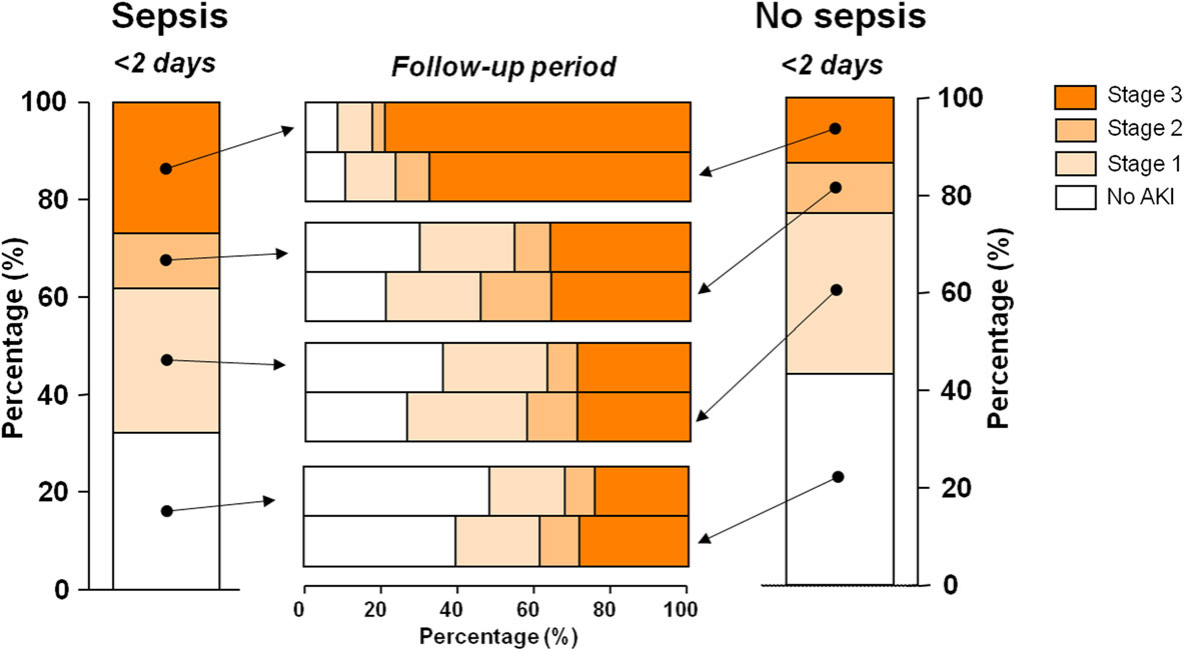
Change in severity of acute kidney injury (AKI) in septic and non-septic critically ill patients over 7 days. Vertical bar graphs represent AKI diagnosed at ICU admission in septic patients (left) and non-septic patients (right), according to AKIN categories: No AKI, stage 1, 2 and 3. Horizontal bar graphs (middle) represent change in AKI stage on day 7 according to AKI admission category. For example, of the 30% (*n* = 575) of septic patients with AKI stage 1, 27% (*n* = 155) remained in the AKI stage 1 group, 36% (*n* = 206) improved to No AKI, 8% (*n* = 45) worsened to stage 2 and 29% (*n* = 169) worsened to stage 3

A total of 10 069 patients were included in the ICON audit [15]; most patients were from ICUs in Europe (54%), Asia (19%) and the Americas (17%). Because of missing serum creatinine and urine output data, 9579 patients (95%) were eligible for the present analyses; 764 patients developed sepsis during the ICU stay (≥ 2 days after admission) and were excluded. The 845 patients with pre-existing CRF were analysed as a separate subgroup. Of the remaining 7970 patients, 1946 (24%) had sepsis on admission. A total of 4727 patients met the criteria for AKI within 72 hours after ICU admission (diagnosis was based on creatinine criteria alone in 14% of patients, urine output criteria in 85% and both criteria in 1%, using the component that led to the worst possible AKIN classification in each case). AKI was more frequent (*n* = 1318 (68%) vs *n* = 3409 (57%), *p* < 0.001) and more severe in patients with sepsis on admission than in those without (Fig. 1). The demographic and clinical characteristics of these patients are presented in Tables 1 and 2.

**Table 1:** Baseline demographic characteristics of patients with and without sepsis, stratified by AKIN stage

	Sepsis				No sepsis			
	No AKI	Stage 1	Stage 2	Stage 3	No AKI	Stage 1	Stage 2	Stage 3
Number of patients, (%)	628 (32.3)	575 (29.5)	219 (11.3)	524 (26.9)	2615 (43.4)	1976 (32.8)	627 (10.4)	806 (13.4)
Age (years), mean (SD)	58.5 (17.6)	61.7 (17.3)^ab^	64.8 (17.8)^ab^	63.3 (15.9)^a^	56.0 (19.1)	59.4 (17.9)	61.1 (17.9)^a^	61.7 (17.8)^a^
Male, *n* (%)	383 (61.8)	352 (62.0)	118 (54.9)	312 (59.8)	1477 (57.4)	1226 (62.6)^a^	376 (60.6)	450 (56.4)
Weight (kg), mean (SD)	70.1 (19.2)	75.3 (21.0)	77.6 (21.4)	76.6 (22.3)	70.5 (16.4)	76.4 (17.7)^a^	80.6 (24.1)^a^	77.9 (23.6)^a^
Height (cm), mean (SD)	167.2 (9.7)	168.0 (10.4)	167.9 (10.2)	167.6 (10.6)	167.1 (10.0)	168.6 (9.5)^a^	168.2 (10.0)^a^	167.5 (10.4)
Severity scores, mean (SD)								
APACHE II	20.6 (7.7)^b^	22.0 (7.8)^ab^	22.3 (7.9)^ab^	26.7 (9.2)^ab^	14.8 (7.5)	15.1 (7.2)	16.1 (8.5)^a^	20.7 (10.9)^a^
Non-renal APACHE II score	19.6 (7.5)^b^	20.9 (7.5)^ab^	21.1 (7.6)^ab^	23.6 (8.4)^ab^	14.1 (7.3)	14.4 (6.9)	15.3 (8.1)^a^	18.6 (10.0)^a^
SOFA	8.1 (3.3)^b^	8.2 (3.4)^ab^	8.7 (3.7)^ab^	11.4 (4.2)^ab^	4.7 (3.4)	4.7 (3.5)	4.8 (3.8)	7.6 (4.9)^a^
Non-renal SOFA	7.4 (3.1)^b^	7.3 (3.1)^ab^	7.6 (3.5)^b^	8.8 (3.7)^ab^	4.0 (3.2)	4.1 (3.2)	4.1 (3.5)	5.5 (4.1)^a^
Type of admission, *n* (%)								
Surgical (non-trauma)	199 (33.8)	171 (31.5)^b^	68 (31.9)	146 (28.9)^a^	953 (38.3)	799 (42.3)^a^	216 (35.9)	209 (28.4)^a^
Medical	355 (60.4)^b^	355 (65.4)^b^	137 (64.3)	348 (68.9)^a^	1277 (51.3)	923 (48.9)	346 (57.6)^a^	482 (65.5)^a^
Trauma	28 (4.8)^b^	16 (2.9)^b^	8 (3.8)	8 (1.6)^ab^	231 (9.3)	159 (8.4)	38 (6.3)^a^	39 (5.3)^a^
Other	6 (1.0)	1 (0.2)^a^	0 (0.0)^a^	3 (0.6)	26 (1.0)	7 (0.4)^a^	1 (0.2)^a^	6 (0.8)
Source of admission, *n* (%)								
Other hospital	62 (9.9)	65 (11.3)	22 (10.0)	59 (11.3)	231 (8.8)	198 (10.0)	55 (8.8)	81 (10.0)
ER/ambulance	229 (36.5)	208 (36.2)	74 (33.8)	156 (29.8)^b^	1047 (40.0)	733 (37.1)	262 (41.8)	335 (41.6)
OR/recovery	88 (14.0)^b^	80 (13.9)^b^	34 (15.5)	65 (12.4)	516 (19.7)	476 (24.1)^a^	107 (17.1)	100 (12.4)^a^
Hospital floor	206 (32.8)^b^	179 (31.1)^b^	63 (28.8)	200 (38.2)^ab^	584 (22.3)	414 (21.0)	148 (23.6)	222 (27.5)^a^
Other	43 (6.8)	43 (7.5)	26 (11.9)	44 (8.4)	237 (9.1)	155 (7.8)	55 (8.8)	68 (8.4)
Reason for admission, *n* (%)								
Neurological	102 (16.2)	66 (11.5)^a^	16 (7.3)^a^	26 (5.0)^a^	438 (16.7)	261 (13.2)^a^	74 (11.8)^a^	59 (7.3)^a^
Respiratory	174 (27.7)^b^	163 (28.3)^b^	54 (24.7)^b^	91 (17.4)^ab^	299 (11.4)	237 (12.0)	100 (15.9)^a^	101 (12.5)
Cardiovascular	143 (22.8)^b^	146 (25.4)^b^	71 (32.4)^a^	200 (38.2)^a^	707 (27.0)	651 (32.9)^a^	203 (32.4)^a^	308 (38.2)^a^
Renal/Ob-gyn	11 (1.8)^b^	15 (2.6)^b^	4 (1.8)	42 (8.0)^a^	99 (3.8)	74 (3.7)	25 (4.0)	73 (9.1)^a^
Haematological	10 (1.6)	17 (3.0)^b^	8 (3.7)	14 (2.7)	37 (1.4)	18 (0.9)	11 (1.8)	17 (2.1)
Digestive/liver	96 (15.3)	106 (18.4)	46 (21.0)^a^	104 (19.8)^ab^	298 (11.4)	287 (14.5)^a^	94 (15.0)^a^	121 (15.0)^a^
Metabolic	21 (3.3)^b^	20 (3.5)^b^	6 (2.7)^b^	19 (3.6)^b^	229 (8.8)	91 (4.6)^a^	37 (5.9)^a^	49 (6.1)^a^
Trauma	49 (7.8)	25 (4.3)^b^	10 (4.6)	8 (1.5)^ab^	337 (12.9)	221 (11.2)	49 (7.8)^a^	48 (6.0)^a^
Other diseases	22 (3.5)^b^	17 (3.0)^b^	4 (1.8)^b^	20 (3.8)	145 (5.5)^b^	98 (5.0)	32 (5.1)	24 (3.0)
Comorbidities, *n* (%)								
COPD	113 (18.0)^b^	91 (15.8)^b^	40 (18.3)	73 (13.9)^b^	256 (9.8)	224 (11.3)	79 (12.6)	86 (10.7)
Cancer	79 (12.6)	67 (11.7)	32 (14.6)	54 (10.3)	273 (10.4)	199 (10.1)	71 (11.3)	69 (8.6)
Metastatic cancer	24 (3.8)	28 (4.9)	9 (4.1)	24 (4.6)	73 (2.8)	68 (3.4)	27 (4.3)	29 (3.6)
Haematologic cancer	33 (5.3)^b^	19 (3.3)^b^	8 (3.7)	26 (5.0)	30 (1.1)	19 (1.0)	11 (1.8)	23 (2.9)^a^
Insulin–DM	44 (7.0)	53 (9.2)	21 (9.6)	60 (11.5)	209 (8.0)	162 (8.2)	46 (7.3)	73 (9.1)
Heart failure, NYHA III–IV	51 (8.1)	57 (9.9)^ab^	22 (10.0)^ab^	69 (13.2)^a^	160 (6.1)	138 (7.0)	41 (6.5)	79 (9.8)^a^
HIV infection	4 (0.6)	4 (0.7)	4 (1.8)	11 (2.1)^ab^	17 (0.7)	5 (0.3)	4 (0.6)	5 (0.6)
Cirrhosis	28 (4.5)^b^	29 (5.0)	9 (4.1)	34 (6.5)	60 (2.3)	63 (3.2)	16 (2.6)	41 (5.1)^a^
Immunosuppression	46 (7.3)^b^	37 (6.4)^b^	16 (7.3)^b^	32 (6.1)^b^	50 (1.9)	36 (1.8)	15 (2.4)	23 (2.9)
Steroid therapy	39 (6.2)^b^	33 (5.7)^b^	13 (5.9)^b^	26 (5.0)^b^	70 (2.7)	50 (2.5)	11 (1.8)	20 (2.5)
Chemotherapy	27 (4.3)^b^	21 (3.7)	7 (3.2)	24 (4.6)	65 (2.5)	45 (2.3)	18 (2.9)	20 (2.5)

*AKIN* Acute Kidney Injury Network, *AKI* acute kidney injury, *SD* standard deviation, n number of patients, *APACHE* Acute Physiology and Chronic Health Evaluation, *SOFA* Sequential Organ Failure Assessment, *ER* emergency room, *OR* operating room, *Ob-gyn* obstetrics–gynaecology, *COPD* chronic obstructive pulmonary disease, *DM* diabetes mellitus, *NYHA* New York Heart Association, *HIV* human immunodeficiency virus

^a^Significant at 5% level vs No AKI in the same group (sepsis or no sepsis)

^b^Significant at 5% level vs no sepsis patients with the same AKIN stage

**Table 2:** Baseline clinical characteristics of patients with and without sepsis, stratified by AKIN stage

	Sepsis				No sepsis			
	No AKI	Stage 1	Stage 2	Stage 3	No AKI	Stage 1	Stage 2	Stage 3
Systemic inflammatory response syndrome								
Temperature, *n* (%)								
< 36 °C	14 (2.3)	19 (3.4)	7 (3.3)	38 (7.4)^a^	59 (2.3)	51 (2.6)	21 (3.4)	69 (8.8)^a^
36–38 °C	415 (67.4)^b^	365 (64.7)^b^	167 (77.7)^b^	341 (66.0)^b^	2033 (80.5)	1654 (84.9)^a^	522 (84.2)^a^	600 (76.4)^a^
> 38 °C	187 (30.4)^b^	180 (31.9)^b^	41 (19.1)^ab^	138 (26.7)^b^	432 (17.1)	243 (12.5)^a^	77 (12.4)^a^	116 (14.8)
Leucocytes, *n* (%)								
< 4 × 10^9^/L	28 (4.6)	22 (3.9)^b^	14 (6.4)^b^	26 (5.0)^b^	79 (3.3)	37 (2.0)	12 (2.0)	18 (2.3)
4–12 × 10^9^/L	247 (40.7)^b^	203 (35.7)^b^	73 (33.5)^b^	157 (30.5)^ab^	1122 (46.4)	925 (49.6)^a^	288 (49.0)	323 (41.8)^a^
> 12 × 10^9^/L	332 (54.7)^b^	343 (60.4)^b^	131 (60.1)^b^	332 (64.5)^ab^	1215 (50.3)	904 (48.4)	288 (49.0)	432 (55.9)^a^
Heart rate (bpm), mean (SD)	112.5 (24.0)^b^	116.1 (26.2)^ab^	111.9 (25.7)^b^	118.5 (26.6)^ab^	104.1 (23.0)	102.3 (23.2)	102.6 (24.5)	106.4 (26.9)^a^
Mean arterial pressure (mmHg), mean (SD)	98.4 (18.6)	97.5 (17.8)	92.1 (17.7)^ab^	91.2 (19.8)^a^	100.3 (19.4)	98.4 (19.0)^a^	98.1 (21.0)^a^	94.1 (21.7)^a^
Respiratory rate (bpm), mean (SD)	25.9 (8.7)^b^	26.6 (9.3)^b^	25.4 (8.8)	26.7 (9.0)^b^	24.2 (7.9)	23.9 (7.6)	24.9 (8.2)	24.7 (8.3)
Patients on ventilator, *n* (%)	434 (69.1)^b^	396 (68.9)^b^	150 (68.5)^b^	378 (72.1)^b^	1089 (41.6)	818 (41.4)	249 (39.7)	364 (45.2)^a^
Blood parameters								
FiO_2_, mean (SD)	59.2 (24.9)^b^	63.9 (25.9)^ab^	63.4 (27.6)^ab^	68.1 (26.9)^ab^	48.2 (24.4)	48.6 (24.2)	49.2 (25.7)	56.4 (28.7)
PaO_2_ (mmHg), mean (SD)	141.2 (72.6)	141.2 (77.8)	138.3 (75.0)	142.2 (81.6)	147.4 (77.5)	143.4 (77.6)	134.3 (75.2)^a^	138.1 (86.3)^a^
PaCO_2_ (mmHg), mean (SD)	45.5 (18.4)^b^	44.8 (19.4)^b^	44.5 (18.2)	44.5 (18.8)^b^	42.0 (15.0)	42.3 (15.6)	43.3 (17.2)	41.3 (17.9)
Bicarbonate (mmol/L), mean (SD)	25.3 (6.2)	24.2 (5.5)	23.6 (6.4)^a^	21.6 (6.3)^a^	24.5 (5.2)	24.1 (4.8)^a^	23.6 (5.4)^a^	21.9 (6.3)^a^
Arterial pH, mean (SD)	7.4 (0.1)	7.4 (0.1)	7.4 (0.1)	7.3 (0.1)	7.4 (0.1)	7.4 (0.1)	7.4 (0.1)	7.3 (0.1)
Creatinine (mg/dl), mean (SD)	1.4 (1.6)^b^	1.5 (1.6)^b^	1.6 (1.5)	3.2 (3.5)^ab^	1.1 (1.4)	1.3 (1.5)	1.4 (1.8)^a^	2.6 (3.3)^a^
Potassium (mmol/L), mean (SD)	4.2 (0.7)	4.3 (0.7)^a^	4.4 (0.8)^a^	4.8 (1.1)^ab^	4.2 (0.7)	4.3 (0.7)^a^	4.3 (0.7)^a^	4.6 (1.0)^a^
Sodium (mmol/L), mean (SD)	140.1 (7.2)	140.6 (6.8)^b^	140.3 (5.9)^b^	139.8 (6.8)	139.7 (5.9)	139.6 (5.7)	139.2 (5.9)	139.4 (6.8)
Urea (mmol/L), median (IQR)	0.6 (0.1–35.0)	5.0 (0.1–39.0)	4.6 (0.1–35.0)	21.0 (0.2–73.0)^ab^	13.0 (0.1–31.0)	16.0 (0.1–37.0)	17.0 (0.1–36.0)	15.0 (0.2–44.0)
Haematocrit (L/L), median (IQR)	33.2 (28.9–38.4)	33.7 (28.5–38.8)	33.0 (28.0–38.0)	32.5 (27.1–37.7)	35.2 (30.0–40.0)	35.6 (30.2–40.6)	36.0 (30.0–41.0)	34.5 (29.0–40.0)
Bilirubin (mg/dl), median (IQR)	0.9 (0.5–2.0)	0.9 (0.5–2.0)	1.1 (0.5–3.0)	1.3 (0.6–3.6)	0.8 (0.5–1.3)	0.8 (0.5–1.4)	0.8 (0.5–1.5)	0.9 (0.5–2.3)
Noradrenaline infusion rate (μg/kg/min), median (IQR)	0.0 (0.0–0.2)	0.0 (0.0–0.2)	0.1 (0.0–0.3)^ab^	0.1 (0.0–0.5)^ab^	0.0 (0.0–0.0)	0.0 (0.0–0.0)	0.0 (0.0–0.0)	0.0 (0.0–0.0)
Inotropic score, median (IQR)	0 (0–19)^b^	0 (0–20)^b^	9 (0–37)^ab^	13 (0–60)^ab^	0 (0–0)	0 (0–0)	0 (0–0)	0.0 (0–10)
Urine output (ml/24 h), median (IQR)	2500 (2000–3280)	1376 (1100–1700)^a^	750 (600–1000) ^a^	355 (120–906)^a^	2500 (2000–3250)	1350 (1100–1666)^a^	750 (600–953)^a^	350 (138–648)^a^

*AKIN* Acute Kidney Injury Network, *AKI* acute kidney injury, *SD* standard deviation, n number of patients, FiO_2_fraction of inspired oxygen, PaO_2_ partial pressure arterial oxygen, PaCO_2_ partial pressure arterial carbon dioxide, *IQR* interquartile range

^a^Significant at 5% level vs No AKI in the same group (sepsis or no sepsis)

^b^Significant at 5% level vs no sepsis patients with the same AKIN stage

### AKI follow-up

By the 7-day follow-up, sepsis patients without AKI within the first 72 hours of ICU admission were less likely to have developed AKI compared to the non-septic population (*n* = 325 (52%) vs *n* = 1582 (60%), *p* < 0.0001). Sepsis patients admitted with AKIN stage 1 or 2 were more likely to have complete recovery of AKI, compared to patients without sepsis for the same AKIN stage (*n* = 206 (36%) vs *n* = 522 (26%) for AKIN stage 1 and *p* < 0.0001; *n* = 65 (30%) vs *n* = 130 (21%) for AKIN stage 2, *p* = 0.005, respectively). However, sepsis patients with AKIN stage 3 were less likely to have recovered to a lower AKIN stage by day 7 than non-septic patients with AKIN stage 3 (*n* = 108 (21%) vs *n* = 258 (32%), *p* < 0.0001). The random intercept model using time points of AKI measurements (admission/follow-up), sepsis (yes/no) and their interaction showed that the estimated parameter for interaction was – 0.62 ± 0.08, *p* < 0.001, confirming that sepsis patients with less severe AKI were more likely to recover renal function within the first week after admission than non-septic patients, whereas sepsis patients with more severe AKI (AKI stage 3) were less likely to recover renal function than non-sepsis patients with AKI stage 3.

### Renal replacement therapy and length of stay

The use of RRT was greater in the septic patients than in the non-septic population (*n* = 380 (20%) vs *n* = 326 (5%), *p* < 0.0001; Additional file 4A)*.* During the first 4 days, haemofiltration was the most frequent form of RRT in sepsis patients and haemodialysis in non-septic patients (Additional file 3: Table S2). RRT use was greatest in patients admitted with AKIN stage 3 in patients with and without sepsis (Table 3). In patients admitted with AKIN stage 3, RRT use was more frequent in patients who remained in stage 3 than in those who improved (Additional file 4B). In patients with AKIN stage 3, RRT was more often initiated in upper-middle and high income-class countries compared to low and lower-middle income-class countries (Additional file 3: Table S3). RRT treatment modality for each region and income class is presented in Additional file 3: Table S4.

**Table 3:** Clinical outcomes

	Sepsis (*n* = 1946)				No sepsis (*n* = 6024)			
	No AKI (*n* = 628)	Stage 1 (*n* = 575)	Stage 2 (*n* = 219)	Stage 3 (*n* = 524)	No AKI (*n* = 2615)	Stage 1 (*n* = 1976)	Stage 2 (*n* = 627)	Stage 3 (*n* = 806)
ICU LOS (days), median (IQR)	7 (2–22)^b^	7 (3–29)^b^	8 (4–54)^ab^	19 (6–60)^ab^	3 (1–5)	3 (2–5)	2 (1–6)^a^	4 (2–60)^a^
Hospital LOS (days), median (IQR)	25 (12–60)^b^	28 (13–60)^b^	49 (14–60)^ab^	60 (22–60)^ab^	10 (6–22)	10 (6–24)	11 (6–35)^a^	20 (7–60)^a^
RRT, *n* (%)	25 (4.0)^b^	33 (5.7)^b^	15 (6.8)^b^	307 (58.6)^ab^	27 (1.0)	22 (1.1)	10 (1.6)	267 (33.1)^a^
ICU mortality, *n* (%)	108 (17.6)^b^	117 (20.6)^ab^	50 (23.5)^ab^	216 (41.5)^ab^	213 (8.4)	138 (7.2)	76 (12.4)^a^	228 (29.2)^a^
Hospital mortality, *n* (%)	154 (25.9)^b^	162 (29.3)^ab^	74 (35.6)^ab^	261 (51.7)^ab^	286 (11.8)	219 (11.9)	102 (17.4)^a^	262 (34.6)^a^

*AKI* acute kidney injury, *ICU* intensive care unit, *LOS* length of stay, *IQR* interquartile range, *RRT* renal replacement therapy, n number of patients

^a^Significant at 5% level vs No AKI in the same group (sepsis or no sepsis)

^b^Significant at 5% level vs no sepsis patients with the same AKIN stage

ICU and hospital LOS was longer for patients admitted with sepsis compared to patients without sepsis (8 (4–60) vs. 3 (2–6), *p* < 0.001 and 38 (14–60) vs 11 (6–32), *p* < 0.001, respectively) and increased with increasing AKI severity (Table 3). ICU and hospital LOS for each region and income class is presented in Additional file 3: Table S5.

### Mortality

Crude mortality rates were higher in patients receiving RRT compared to those without RRT (ICU mortality, sepsis *n* = 150 (40%) vs *n* = 341 (22%) and no sepsis *n* = 91 (29%) vs *n* = 564 (10%); in-hospital mortality, sepsis *n* = 180 (49%) vs *n* = 71 (32%) and no sepsis *n* = 105 (34%) vs *n* = 764 (14%), all *p* < 0.001). Of those receiving RRT, in-hospital mortality was higher in non-septic patients receiving haemofiltration compared to haemodialysis, whereas in the septic group there were no differences between the modalities (Additional file 3: Table S6).

Crude in-hospital mortality rates for patient with and those without sepsis were 2–3-fold higher in patients with AKIN stage 3 compared to patients without AKI and were higher for each AKIN stage compared to no AKI in patients with sepsis (Table 3). In patients with AKIN stage 3, in-hospital mortality was comparable for each income class (Additional file 3: Table S3).

**Fig. 2:**
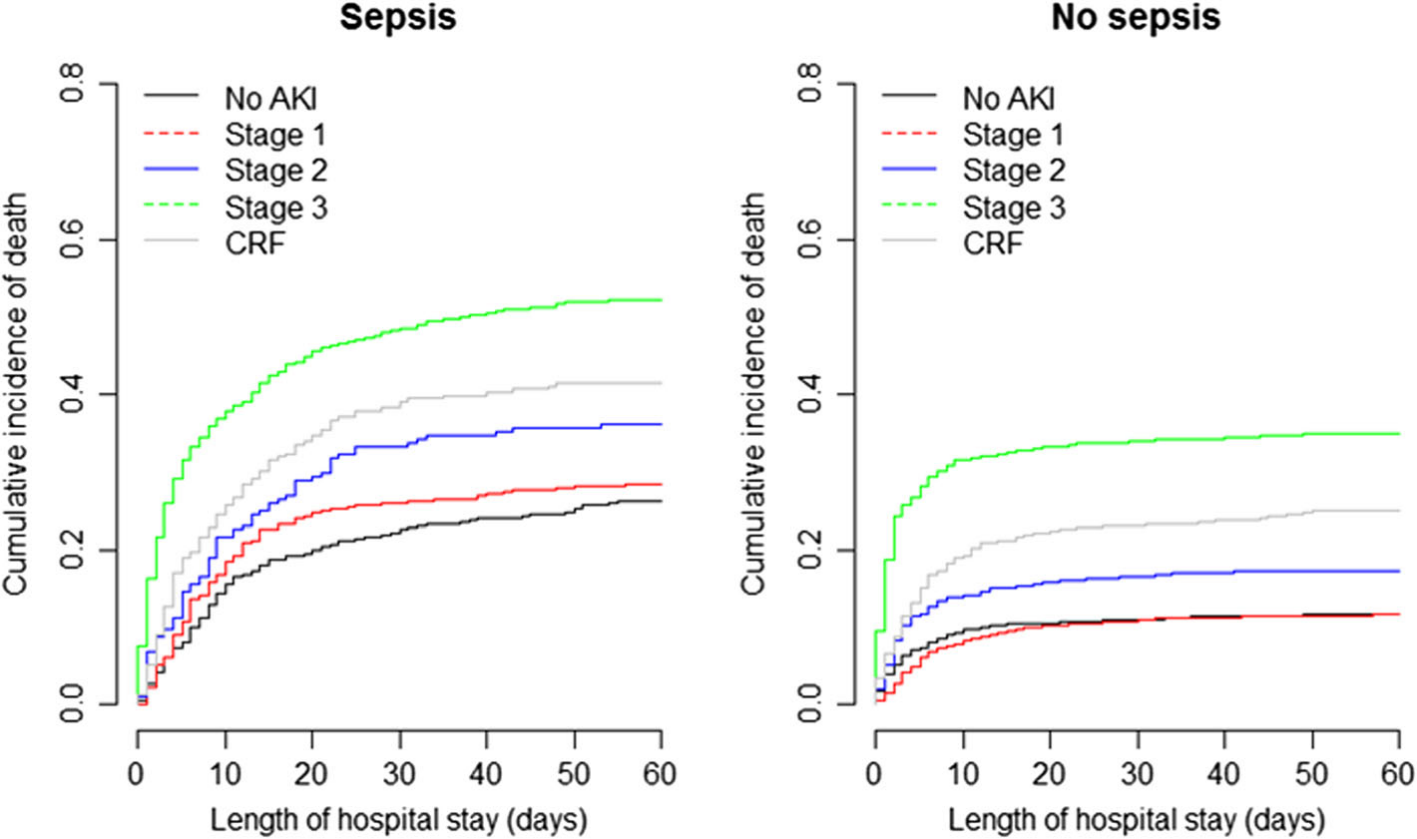
Cumulative incidence functions of death in critically ill patients with (left) and without (right) sepsis according to acute kidney injury (AKI) stage on admission and presence of comorbid chronic renal failure (CRF)

In patients with sepsis, crude in-hospital mortality was greatest in those admitted with AKI stage 3 and AKI stage 2 who had not recovered by day 7 (Additional file 5); in patients without sepsis, this relationship was only present in patients with AKI stage 3. AKI stage 3 patients who fully recovered renal function had a 13% (septic patients) and 20% (non-septic patients) lower in-hospital mortality compared to those who remained in stage 3 at day 7. However, in-hospital mortality in these patients was still twice as high compared to patients recovering from AKI stages 2 and 1 (Additional file 3). Cumulative survival curves are shown in Fig. 2. AKI stages 2 and 3 were associated with significantly higher hazard ratios of crude ICU and hospital mortality in septic and non-septic patients compared to no AKI (Table 4). When adjusted for covariates, AKI stage 3 was still associated with ICU and in-hospital mortality in both patient groups (with and without sepsis) and AKI stage 2 was associated with in-hospital mortality in patients without sepsis (Table 4).

**Table 4:** Hazard ratios for mortality

	Sepsis		No sepsis	
	Crude HR (95% CI)	Covariate-adjusted HR (95% CI)	Crude HR (95% CI)	Covariate-adjusted HR (95% CI)
ICU mortality				
Stage 1	1.16 (0.91–1.49)^c^	1.03 (0.80–1.33)^c^	0.84 (0.68–1.04)^c^	0.83 (0.67–1.02)^c^
Stage 2	1.39 (1.01–1.92)^a^	1.30 (0.94–1.80)	1.49 (1.15–1.95)^ac^	1.21 (0.94–1.57)
Stage 3	2.77 (2.22–3.46)^abc^	1.90 (1.48–2.43)^a^	4.06 (3.36–4.91)^ac^	2.25 (1.83–2.76)^ac^
CRF	1.67 (1.26–2.22)^a^	1.54 (1.13–2.10)^a^	2.21 (1.74–2.81)^a^	1.38 (1.07–1.77)^a^
Hospital mortality				
Stage 1	1.12 (0.91–1.39)^c^	0.99 (0.79–1.23)^c^	0.98 (0.82–1.17)^c^	0.94 (0.79–1.12)^c^
Stage 2	1.46 (1.12–1.89)^a^	1.27 (0.96–1.68)	1.51 (1.20–1.90)^ac^	1.26 (1.01–1.58)^a^
Stage 3	2.47 (2.04–2.99)^abc^	1.74 (1.41–2.16)^a^	3.61 (3.03–4.29)^ac^	2.26 (1.87–2.72)^ac^
CRF	1.76 (1.39–2.22)^a^	1.54 (1.18–2.01)^a^	2.28 (1.85–2.80)^a^	1.44 (1.15–1.81)^a^

No AKI is the reference category. Hazard ratios were estimated by competing risk regression models

*HR* hazard ratio, *CI* confidence interval, *ICU* intensive care unit, *CRF* chronic renal failure

^a^Significant at 5% level vs No AKI in the same group (sepsis or no sepsis)

^b^Significant at 5% level vs non-sepsis patients with the same AKIN classification

^c^Significant at 5% level vs CRF in the same group (sepsis or no sepsis)

### Patients with chronic renal failure

Of the 845 patients with a medical history of CRF, 263 (31%) had sepsis on admission. Baseline demographic and clinical characteristics are presented in Additional file 3: Tables S7 and S8. Use of RRT and ICU and in-hospital LOS and mortality are presented in Additional file 3: Table S9. Patients with CRF had lower ICU and in-hospital mortality rates than those with AKI stage 3 patients (sepsis *p* = 0.017; non-sepsis: *p* < 0.001) (Fig. 2). When adjusted for covariates, these differences disappeared in patients with sepsis, but remained in patients without sepsis (Table 4).

## Discussion

The data obtained from this large international multicentre audit confirm previously reported prevalences of AKI and that the condition is more common in patients with sepsis than in those without [4, 7–9, 24]. Our data on the changes in severity of AKI mirror those of the FINAKKI study, in which 86% of critically ill patients admitted with AKIN stage 3 remained in this most severe stage [4]. Our results also corroborate those from a study by Hoste et al. [7], in which more than half of the AKI-Risk (similar to AKIN stage 1) patients and one-third of the AKI-Injury (similar to AKIN stage 2) patients deteriorated during the ICU stay. However, neither of these studies differentiated between patients with and without sepsis. Moreover, although it is well known that increasing severity of AKI is associated with a higher mortality [7], we also show that transition to a lower AKIN stage within 7 days is associated with an improvement in survival. Nevertheless, once AKIN stage 3 is reached, hospital mortality remains high, even if there is apparent full renal recovery within 7 days after ICU admission.

### AKI in patients with and without sepsis

We observed that septic patients with less severe AKI were more likely to recover renal function during the first week after admission than non-septic patients with the same initial degree of AKI, whereas septic patients admitted with AKIN stage 3 were less likely to recover to a lower AKIN stage. Importantly, sepsis and non-sepsis groups are not necessarily similar at baseline, as patients with sepsis are more likely to have early, evolving AKI, and so changes in illness trajectory may reflect a temporal bias, rather than the influence of the underlying disease. Also, if the bar is set at the creatinine level at admission, it may be easier for patients who have a lower creatinine level pre admission to get back to this level. Clearly, there also are differences in the distinct pathogenesis of septic versus non-septic AKI. Although a variety of mechanisms are involved in the development of AKI [25], AKI in patients with sepsis may predominantly be the consequence of the dysregulated inflammatory response to an infection, resulting in renal inflammation, microcirculatory dysfunction and cell bioenergetic adaptive responses [13]. Clearing underlying infection may be more likely to help AKI resolution in milder cases of sepsis-associated AKI than in more severe stages. Conversely, non-septic AKI may be related more to extensive damage to the micro-architecture of the kidney, requiring prolonged periods for repair and renal recovery. In support of this suggestion, autopsy investigations did not reveal widespread tubular damage or cell death in septic AKI [26].

### Influence of RRT and transition to another AKIN stage

In this study, RRT was mostly started within the first 2 days after ICU admission and predominantly in patients admitted with AKIN stage 3 who did not recover during the first week after admission. It is currently unclear whether or not early initiation of RRT may improve patient outcome [27, 28] despite two large clinical trials [29, 30] and further study, such as the ongoing STARRT-AKI trial (ClinicalTrials.gov NCT02568722), is necessary to elucidate the effects of RRT timing on outcomes and to guide clinical decisions related to the initiation of RRT.

Although in-hospital mortality was lower in patients with renal recovery, septic and non-septic AKIN stage 3 patients who had recovered full renal function by day 7 still had a 2-fold higher in-hospital mortality rate than patients recovering from AKI stage 2 or 1 or patients without AKI. In addition, our data confirm that mortality is higher in patients who receive RRT compared to those who do not [31]. Our hypothesis-generating data could encourage a novel approach to risk stratification in AKI for current therapy, future investigations and clinical therapeutic trials. For example, in the absence of life-threatening complications, it may be prudent to delay RRT, as a large number of patients, especially in the lower AKIN stages, recovered full renal function. Indeed, use of RRT may delay recovery of renal function [32]. While resuscitation strategies appear not to influence the development of AKI in patients with septic shock [33], secondary prevention, by mitigating further toxic and haemodynamic renal insults, may represent an important therapeutic opportunity in this population. Also, models of ICU mortality and prognostic scores could likely be refined by considering the dynamic changes in renal function over time.

### Limitations of this study

Our study has several limitations. Firstly, we lack information about secondary or post-day 7 renal insults that may have occurred as a result of hypotension, adverse drug reactions or inappropriate blood pressure targets. These factors can play an important role in the deterioration or improvement of AKI. Secondly, renal function was assessed using the AKIN criteria, a widely accepted and validated classification system for AKI in the critically ill [17], which we adjusted because urine output was only available per 24-hour period. As a consequence of using AKIN categories and not absolute creatinine values, smaller changes could be missed. Importantly, patients admitted to the ICU with ongoing AKI may be missed using these criteria and/or the severity of AKI was not correctly identified because pre-ICU admission reference creatinine values were not available, as is frequently the case in clinical ICU practice. This may limit the determination of the clinical consequences of deterioration or resolution of AKI, and may hamper comparison between sepsis and non-sepsis patients. Thirdly, we have no data enabling analysis of longer term consequences of AKI and progression to chronic kidney disease or AKI relapse in patients who had recovered normal renal function by day 7. These long-term consequences, stratified by evolution of AKI, represent an important area for future investigations. Fourthly, although the worldwide inclusion of patients provides valuable insight into the global burden of AKI, the causes of AKI vary by country and economic status [34]. Also, resources may differ in various regions around the world, which could influence the clinical outcome of AKI patients. However, we showed that although the use of dialysis in AKIN stage 3 patients was higher in upper-middle and high-income countries compared to low and lower-middle-income countries, in-hospital mortality rates were comparable across income classes in these patients. Finally, some centres included a limited number of patients, possibly leading to sampling bias, and the voluntary participation of ICUs may have resulted in compliance bias. Nevertheless, this is a prospective study in which a large number of patients was included from all over the world with daily assessment of renal function, enabling a useful comparison of the prevalence and evolution of AKI and its consequences among patients with and without sepsis.

## Conclusion

AKI is more frequent, more severe, less likely to resolve once AKIN stage 3 has been reached and associated with higher mortality rates in patients with sepsis than in those without*.* Differences in the phase of AKI development and aetiology of AKI likely account for these observations in sepsis and non-sepsis patients. Deterioration to a more severe stage of AKI negatively influences clinical outcome, while improvement is associated with increased survival. These results emphasize the severity of the disease, which represents a tremendous burden for both patients and society.

## Additional files

Additional file 1: Online supplement methods: complete methods including study population, data collection, definitions, quality management and statistical analysis (DOCX 37 kb)

Additional file 2: Alphabetical list of participating centres by region and country (DOCX 26 kb)

Additional file 3: **Table S1.** Adjusted AKIN criteria for diagnosis of AKI. **Table S2.** RRT modality per day. **Table S3.** RRT and mortality per region and GNI. **Table S4.** RRT modality per region and GNI. **Table S5.** Length of stay per region and GNI. **Table S6.** Mortality per RRT modality. **Table S7.** Baseline demographic characteristics of patients with CRF, with and without sepsis. **Table S8.** Baseline clinical characteristics of patients with CRF, with and without sepsis. **Table S9.** Outcome of patients with CRF at admission (DOCX 48 kb)

Additional file 4: **Figure S1.** RRT in critically ill patients with and without sepsis. A: Percentage of patients receiving RRT per day per AKIN stage at admission. B: RRT incidence per AKIN stage at follow-up. Admission category presented on *x* axis. Patients followed-up until day 7 to determine whether they improved to a lower AKIN stage or deteriorated to a worse AKIN stage, presented on *z* axis. In case of discharge or death, data were imputed by last observation carried forward (PNG 43 kb)

Additional file 5: **Figure S2.** Hospital mortality in septic and non-septic critically ill patients during follow-up. At admission (first 72 hours in ICU), patients were categorized as No AKI and AKIN stages 1, 2 and 3, presented on *x* axis. Patients followed-up until day 7 to determine whether they improved to a lower AKIN stage or deteriorated to a more severe AKIN stage, presented on *z* axis. Hospital mortality determined for groups in which patients improved or deteriorated during follow-up. Patients with AKIN stage 3 at admission and at follow-up show highest mortality rate. Worsening of AKI is associated with higher mortality unrelated to AKI category at ICU admission. Graph also shows that patients with AKIN stage 3 at admission who improve to No AKI at follow-up still have twice as high mortality rates compared to patients with No AKI at admission and follow-up (PNG 30 kb)

## Abbreviations

AKI: Acute kidney injury
AKIN: Acute Kidney Injury Network
APACHE: Acute Physiology and Chronic Health Evaluation
CRF: Chronic renal failure
GNI: Gross national income
HR: Hazard ratio
ICON: Intensive Care Over Nations
ICU: Intensive care unit
IQR: Interquartile range
LOS: Length of stay
RRT: Renal replacement therapy
SD: Standard deviation
SOFA: Sepsis-related Organ Failure Assessment
