# Supplementary material for: A worldwide multicentre evaluation of the influence of deterioration or improvement of acute kidney injury on clinical outcome in critically ill patients with and without sepsis at ICU admission: results from The Intensive Care Over Nations audit
Source: Crit Care. 2018 Aug 3;22:188. doi: 10.1186/s13054-018-2112-z (PMC6091052; doi:10.1186/s13054-018-2112-z)
Supplement: Supplementary file 1 — Online supplement methods: complete methods including study population, data collection, definitions, quality management and statistical analysis (DOCX 37 kb) [file 13054_2018_2112_MOESM1_ESM.docx]

**Complete methods**

**Study population and data collection**

This was a sub study of the Intensive Care Over Nations (ICON) audit, a multicentre, worldwide audit conducted between May 8th and May 18th, 2012, to collect data on characteristics, including organ dysfunction, infection and outcomes, of adult critically ill patients worldwide. Full details of the methodology have been provided previously.[1] ICUs were recruited by open invitation and participated on a voluntary basis with no financial incentive. A list of participating centres is provided in Additional file 6. In each institution, the study was approved by the institutional research ethics committee in accordance with local ethical regulations. Informed consent was not required due to the observational and anonymous nature of data collection.

The participating centres prospectively collected data from all adult patients (>16 years) admitted to the ICU during the study period, except those admitted for less than 24 h for routine postoperative surveillance. Readmissions of previously included patients were not included. Data were collected daily during the ICU stay for up to 28 days and patients were followed up for outcome data until hospital discharge or death for a maximum of 60 days. Participating centres submitted data using a secured electronic case report form.[1] Data collected included demographic and comorbidity data, microbiological and clinical infections, use of antibiotics, and clinical and laboratory data. The acute physiology and chronic health evaluation (APACHE) II[2] and non-renal APACHE II were calculated from the worst values taken during the first 24 h after admission (day 0). The sequential organ failure assessment (SOFA)[3] and non-renal SOFA score were calculated daily. For the purposes of this substudy, only patients with full creatinine and urine output data were included. Patients who developed sepsis during the ICU stay (≥ 2 days after admission) were excluded because we wanted to evaluate the impact of sepsis on admission on AKI outcomes.

**Definitions**

Sepsis was defined as the occurrence of at least one failing organ (Sepsis-related Organ Failure Assessment (SOFA) score > 2 for respective organ system), combined with the presence of infection, as defined according to the International Sepsis Forum [4]. The presence of AKI was evaluated using the Acute Kidney Injury Network (AKIN) criteria [5]. AKI stages were determined according to the AKIN criteria, using the largest increase between two values of serum creatinine obtained maximally 48 hours apart within a maximum period of 72 hours after ICU admission or the urine output for the 24-hour period after ICU admission. As urine output was only recorded per 24 hour period in ICON, we adjusted the stage 1 and 2 urine output criteria (Additional file 1, Table S1). The criterion (urine output or serum creatinine) that led to the worst possible AKIN classification was used in each case. We chose to use the AKIN criteria rather than the more recent KDIGO criteria, because in the KDIGO definition of AKI, the creatinine criterion relies on the percentage change over a period of 7 days (rather than 48 hours for the AKIN criteria) and our aim was to demonstrate the effect of deteriorating or resolving AKI during the first 7 days after ICU admission on outcome. To assess AKI deterioration or improvement, the last serum creatinine or urine output data available before discharge or death up to day 7 were used to define the AKIN stage. This AKIN stage was compared to the initial stage, and patients were identified as having deteriorated, improved, or remained the same. Complete recovery was defined as improvement from AKIN stage 1, 2 or 3 to the No AKI category. The inotropic score was calculated to express the use of vasoactive/vasopressor agents, as described elsewhere [6]. The inotropic score was calculated to express the use of vasoactive/vasopressor agents, as described elsewere.[6]

Patients with comorbid chronic renal failure (CRF), indicated on the original ICON case report form by the ICON investigator, were analyzed as a separate subgroup.

**Quality management**

All participating centres had access to a secured website throughout the study, on which detailed instructions about the aim of the study, data collection and definitions were provided. A coordinating centre (Erasme Hospital) answered any questions during data collection on a case-by-case basis. Validity of the data was checked upon data entry on the eCRF, including checks for plausibility within and between variables. The coordinating centre further reviewed all data for plausibility and availability of the outcome parameter (death or hospital discharge). Any uncertainties were clarified by consulting the centre in question. There was no on-site monitoring.

**Statistical analysis**

All statistical analyses were conducted in the Department of Intensive Care of Erasme Hospital. For the purpose of this study, the world was divided into nine geographical regions, and individual countries were classified into three income groups in accordance with the gross national income (GNI) per person [8]. Data are expressed as mean and standard deviation (SD), median with interquartile range [IQR, first and third] or numbers and percentages. For continuous variables, normality assumption checking was performed by inspection of residual and normal plots and by using the Kolmogorov-Smirnov test. Difference testing between groups was performed using the Generalized Linear Models procedure, Kruskal-Wallis test, Student’s T test, Mann-Whitney test, χ² test, or Fisher’s exact test, as appropriate.

To describe the prevalence of patients receiving RRT up to day 7, data were imputed by the last observation carried forward in case of discharge or death. If a patient died before day 60, ICU and hospital lengths of stay (LOS) were set to 60 days.

Data were censored at day 60. In addition, discharge of a patient was considered as a competing risk factor for the occurrence of death. A competing risk regression model [9] was used to estimate crude and adjusted hazard ratios (HRs) and their 95% CIs for ICU and hospital mortality according to AKI stage or CRF group. To determine the adjusted relative risk of in-ICU or in-hospital death, we developed a multivariable competing risk proportional hazard regression model, stratified according to the presence or not of sepsis. Other confounding variables considered included age, sex, Acute Physiology and Chronic Health Evaluation (APACHE) II score without age and renal components, type of admission, source of admission, reason for admission, the need for mechanical ventilation or RRT on admission to the ICU, comorbidities, and the inotrope score. We also adjusted for ICU and hospital-related organizational factors including type of hospital, ICU specialty, total number of ICU patients in 2011, and number of staffed ICU beds. Geographic region and GNI were also considered. Collinearity between variables was excluded before modelling and the time-dependent covariate method was used to check the proportional hazard assumption of the model. The cumulative incidence functions of death according to AKIN stage at admission or CRF were plotted and Gray’s test was used to test cause-specific death differences [10, 11]. To quantify the association between the direction of the evolution of the AKI and sepsis, a cumulative link mixed-effects ordinal response model with logit link function was fitted. The random intercept model was used. The longitudinal ordinal response variable, AKIN stage, was the main dependent. The explanatory variables were the time points of AKIN stage assessment (admission/follow-up), sepsis (yes/no) and their interaction [12, 13]. A two-sided p value less than 0.05 was considered statistically significant. Data were analyzed using IBM® SPSS® Statistics software, version 23 for Windows (IBM, Armonk, NY) and R software, version 2.10.1 (CRAN project).

**References**

1. Vincent JL, Marshall JC, Namendys-Silva SA, Francois B, Martin-Loeches I, Lipman J, Reinhart K, Antonelli M, Pickkers P, Njimi H *et al*: **Assessment of the worldwide burden of critical illness: the intensive care over nations (ICON) audit**. *Lancet Respir Med* 2014, **2**(5):380-386.

2. Knaus WA, Draper EA, Wagner DP, Zimmerman JE: **APACHE II: a severity of disease classification system**. *Critical care medicine* 1985, **13**(10):818-829.

3. Vincent JL, Moreno R, Takala J, Willatts S, De Mendonca A, Bruining H, Reinhart CK, Suter PM, Thijs LG: **The SOFA (Sepsis-related Organ Failure Assessment) score to describe organ dysfunction/failure. On behalf of the Working Group on Sepsis-Related Problems of the European Society of Intensive Care Medicine**. *Intensive care medicine* 1996, **22**(7):707-710.

4. Calandra T, Cohen J, International Sepsis Forum Definition of Infection in the ICUCC: **The international sepsis forum consensus conference on definitions of infection in the intensive care unit**. *Crit Care Med* 2005, **33**(7):1538-1548.

5. Mehta RL, Kellum JA, Shah SV, Molitoris BA, Ronco C, Warnock DG, Levin A, Acute Kidney Injury N: **Acute Kidney Injury Network: report of an initiative to improve outcomes in acute kidney injury**. *Crit Care* 2007, **11**(2):R31.

6. Cruz DN, Antonelli M, Fumagalli R, Foltran F, Brienza N, Donati A, Malcangi V, Petrini F, Volta G, Bobbio Pallavicini FM *et al*: **Early use of polymyxin B hemoperfusion in abdominal septic shock: the EUPHAS randomized controlled trial**. *JAMA* 2009, **301**(23):2445-2452.

7. Bellomo R, Ronco C, Kellum JA, Mehta RL, Palevsky P, Acute Dialysis Quality Initiative w: **Acute renal failure - definition, outcome measures, animal models, fluid therapy and information technology needs: the Second International Consensus Conference of the Acute Dialysis Quality Initiative (ADQI) Group**. *Critical care* 2004, **8**(4):R204-212.

8. Mehta RL, Bouchard J, Soroko SB, Ikizler TA, Paganini EP, Chertow GM, Himmelfarb J, Program to Improve Care in Acute Renal Disease Study G: **Sepsis as a cause and consequence of acute kidney injury: Program to Improve Care in Acute Renal Disease**. *Intensive Care Med* 2011, **37**(2):241-248.

9. Fine JP, Gray RJ: **A Proportional Hazards Model for the Subdistribution of a Competing Risk**. *Journal of the American Statistical Association* 1999, **94**(446):496-509.

10. Southern DA, Faris PD, Brant R, Galbraith PD, Norris CM, Knudtson ML, Ghali WA, Investigators A: **Kaplan-Meier methods yielded misleading results in competing risk scenarios**. *J Clin Epidemiol* 2006, **59**(10):1110-1114.

11. Gray RJ: **A Class of K-Sample Tests for Comparing the Cumulative Incidence of a Competing Risk**. *The Annals of Statistics* 1988, **16**(3):1141-1154.

12. Hedeker D: **Methods for Multilevel Ordinal Data in Prevention Research**. *Prev Sci* 2015, **16**(7):997-1006.

13. Hedeker D, Gibbons RD: **Mixed-effects regression models for ordinal outcomes. In: Longitudinal Data Analysis.**: John Wiley & Sons; 2006.
