# Supplementary material for: A worldwide multicentre evaluation of the influence of deterioration or improvement of acute kidney injury on clinical outcome in critically ill patients with and without sepsis at ICU admission: results from The Intensive Care Over Nations audit
Source: Crit Care. 2018 Aug 3;22:188. doi: 10.1186/s13054-018-2112-z (PMC6091052; doi:10.1186/s13054-018-2112-z)
Supplement: Supplementary file 2 — Alphabetical list of participating centres by region and country (DOCX 26 kb) [file 13054_2018_2112_MOESM2_ESM.docx]

**Alphabetical list of participating centres by region and country**

***Africa***
*Angola*: Clinica Sagrada Esperança (E Tomas)
*Democratic Republic of Congo*: Cliniques Universitaires De Kinshasa (E Amisi Bibonge)
*Morocco*: Chu Ibn Rochd Casablanca (B Charra); Ibn Sina Hospital (M Faroudy)
*South Africa*: Chris Hani Baragwanath Academic Hospital (L Doedens); Grey's Hospital (Z Farina); Sandton Medi Clinic (D Adler); Tygerberg Hospital (C Balkema); Union Hospital Alberton (A Kok)
*Tunisia*: Bizerte Hospital (S Alaya); Military Hospital of Tunis (H Gharsallah)

***East Europe***
*Albania*: National Trauma Centre and Military Hospital, Tirana (D Muzha)
*Bulgaria*: Alexandrovska University Hospital (A Temelkov); Emergency University Hospital 'Pirogov' (G Georgiev); Tokuda Hospital Sofia (G Simeonov); Uh St Ekaterina Sofia (G Tsaryanski); University Hospital for Obstetrics and Gynaecology (S Georgiev); University Hospital Sveta Marina - Varna (A Seliman)
*Croatia*: General Hosp. Sibenik (S Vrankovic); University Hospital Centre "Sestre Milosrdnice" (Z Vucicevic); University Hospital Centre Zagreb (I Gornik); University Hospital for Infectious Diseases (B Barsic); University Hospital Dubrava (I Husedzinovic)
*Czech Republic*: Centre of Cardiovascular and Transplant Surgery (P Pavlik); Charles University Hospital (J Manak); IKEM, Prague (E Kieslichova); KNTB Zlín A.S. (R Turek); Krajska Nemocnice Liberec (M Fischer); Masarykova Nemocnice V Usti Nad Labem (R Valkova); St. Anne's University Hospital Brno (L Dadak); University Hospital Haradec Králové (P Dostal); University Hospital Brno (J Malaska); University Hospital Olomouc (R Hajek); University Hospital Plzen (A Židková); Charles University Hospital Plzen (P Lavicka)
*Estonia*: Tartu University Hospital (J Starkopf)
*Georgia*: Critical Care Medicine Institute (Z Kheladze); Jo Ann Medical Centre (M Chkhaidze); Kipshidze Central University Hospital (V Kaloiani)
*Hungary*: Dr. Kenessey Albert Hospital (L Medve); Fejér County St George Teaching Hospital (A Sarkany); Flor Ferenc County Hospital (I Kremer); Jávorszky Ödön Hospital (Z Marjanek); Peterfy Hospital Budapest (P Tamasi)
*Latvia*: Infectology Centre of Latvia (I Krupnova); Paul Stradins Clinical University Hospital (I Vanags); Riga East Clinical University Hospital (V Liguts)
*Lithuania*: Hospital of Lithuanian University of Health Sciences Kauno Klinikos (V Pilvinis); Vilnius University Hospital (S Vosylius); Vilnius University Hospital "Santariskiu Clinics", HSICU (G Kekstas); Vilnius University Hospital Santariskiu Clinics, CICU (M Balciunas)
*Poland*: Csk Mswia (J Kolbusz); Medical University (A Kübler); Medical University Of Wroclaw (B Mielczarek); Medical University Warsaw (M Mikaszewska-Sokolewicz); Pomeranian Medical University (K Kotfis); Regional Hospital in Poznan (B Tamowicz); Szpital Powiatowy W Ostrowi Mazowieckiej (W Sulkowski); University Hospital, Poznam (P Smuszkiewicz); Wojewódzki Szpital Zakazny (A Pihowicz); Wojewódzkie Centrum Medyczne (E Trejnowska)
*Romania*: Emergency County Hospital Cluj (N Hagau); Emergency Institute for Cardiovascular Diseases (D Filipescu); Fundeni Clinical Institute (G Droc); Galati Hospital (M Lupu); Inbi "Prof. Dr. Matei Bals" (A Nica); Institute of Pulmonology Marius Nasta (R Stoica); Institutul Clinic Fundeni (D Tomescu); Sfantul Pantelimon Hospital (D Constantinescu); Spitalul Cf 2 Bucuresti (G Valcoreanu Zbaganu); “Luliu Hatieganu” University of Medicine and Pharmacy,

Teaching Hospital of Infectious Diseases, Cluj-Napoca (A Slavcovici)
*Russia*: City Clinical Hospital No 40 (V Bagin); City Hospital No 40 (D Belsky); Clinical Hospital N.A. N.V.Solovyev (S Palyutin); Emergency Research Institute N.A. Djanelidze (S Shlyapnikov); Federal Research Centre Paediatric Haematology, Oncology and Immunology (D Bikkulova); Krasnoyarsk State Medical University, Krasnoyarsk Regional Hospital (A Gritsan); Medical Association "Novaya Bolnitsa" (G Natalia); Military Medical Academy (E Makarenko); Novosibirsk Medical University (V Kokhno); Omsk Regional Clinical Hospital (A Tolkach); Railway Hospital of Khabarovsk (E Kokarev); St Alexy Hospital (B Belotserkovskiy); State District Hospital (K Zolotukhin); Vishnevsky Institute of Surgery (V Kulabukhov)
*Serbia*: Clinic for Cardiac Surgery, Clinical Centre of Serbia (L Soskic); Clinic for Digestive Surgery, Clinical Centre Serbia (I Palibrk); Clinic for Vascular Surgery, Clinical Centre Nis (R Jankovic); Clinical Centre of Serbia (B Jovanovic); Clinical Centre of Serbia (M Pandurovic); Emergency Centre, Clinical Centre of Belgrade (V Bumbasirevic); General University Hospital (B Uljarevic); Military Medical Academy (M Surbatovic); Urology Hospital (N Ladjevic)
*Slovakia*: District Hospital (G Slobodianiuk); Faculty Hospital (V Sobona); University Hospital Bratislava-Hospital Ruzinov ICU (A Cikova); University Hospital Ruzinov Bratislava (A Gebhardtova)

***East & Southeast Asia***
*China*: A Tertiary Hospital (C Jun); Affiliated Hospital of Medical College Qingdao Universty (S Yunbo); Beijing Cancer Hospital ,Beijing Institute for Cancer Research (J Dong); Beijing Chaoyang Hospital (S Feng); Beijing Friendship Hospital (M Duan); Beijing Tongren Hospital Affiliate of Capital Medical University (Y Xu); Beijing University People's Hospital (X Xue); Beijing Luhe Hospital (T Gao); Cancer Hospital, Chinese Academy of Medical Sciences (X Xing); China Academy of Chinese Medical Sciences Guang 'An Men Hospital (X Zhao); Chuxiong, Yunnan Province, People's Hospital (C Li); Donge County People's Hospital of Shandong Province (G Gengxihua); Fu Wai Hospital, Chinese Academy of Medical Sciences (H Tan); Fujian Provincial Hospital (J Xu); Fuxing Hospital, Capital Medicine University (L Jiang); Guangdong General Hospital (Q Tiehe); Henan Provincial People's Hospital (Q Bingyu); Xian Jiaotong University College of Medicine (Q Shi); Kunming Third People's Hospital (Z Lv); Lanzhou University Second Hospital (L Zhang); No 309th Hospital (L Jingtao); No.1 Hospital of China Medical University (Z Zhen); Peking University Shougang Hospital (Z Wang); Peking University Third Hospital (T Wang); Pla Navy General Hospital (L Yuhong); Qilu Hospital Shandong University (Q Zhai); Ruijin Hospital Affiliated Medical School of Jiaotong University, Shanghai (Y Chen); Shandong Provincial Hospital (C Wang); Shanghai 10th People's Hospital (W Jiang); Shanghai First People's Hospital (W Ruilan); Sichuan Provincial People's Hospital (Y Chen); Sichuan Provincial People's Hospital (H Xiaobo); Sir Run Run Shaw Hospital (H Ge); The Affiliated of Guiyang Medical College (T Yan); The Fifth People's Hospital of Shanghai, Fudan University (C Yuhui); The First Affiliated Hospital of Dalian Medical University (J Zhang); The First Affiliated Hospital of Suzhou University (F Jian-Hong); The First Affiliated Hospital of Xinjiang Medical University (H Zhu); The First Hospital of Jilin University (F Huo); The First Hospital of Jilin University (Y Wang); The First People's Hospital of Kunming (C Li); The General Hospital of Shenyang Military Region ,China (M Zhuang); The People’s Hospital of Cangzhou (Z Ma); The Second Hospital of Jilin University (J Sun); The Second People’s Hospital of Liaocheng City Shandong Province (L Liuqingyue); The Third Xiangya Hospital (M Yang); Tongde Hospital of Zhejiang Province (J Meng); Tongji University Shanghai East Hospital (S Ma); West China Hospital, Scu (Y Kang); Wuhan Centre Hospital (L Yu); Xiangya Hospital, Changsha, Hunan Province, China (Q Peng); Yantai Yuhuangding Hospital (Y Wei); Yantaishan Hospital, Shandong Province (W Zhang); Zhejiang Provincial People's Hospital (R Sun)
*Hong Kong* (China): Pamela Youde Nethersole Eastern Hospital (A Yeung); Princess Margaret Hospital (W Wan); Queen Elizabeth Hospital (K Sin); United Christian Hospital of Hong Kong SAR (K Lee)
*Indonesia*: Anestesi (M Wijanti); Pku Muhammadiyah Bantul, Yogyakarta (U Widodo); Rd Mattaher Hospital Jambi (H Samsirun); Rumah Sakit Pantai Indah Kapuk (T Sugiman); Sardjito Hospital (C Wisudarti); School of Medicine Unpad - Hasan Sadikin Hospital (T Maskoen)
*Japan*: Chiba Hokusoh Hospital, Nippon Medical School (N Hata); Chiba University Hospital (Y Kobe); Fujita Health University School of Medicine (O Nishida); Japanese Red Cross Maebashi Hospital (D Miyazaki); Jichi Medical University Hospital (S Nunomiya); Jikei University School of Medicine (S Uchino); Kimitsu Chuo Hospital (N Kitamura); Kochi Medical School (K Yamashita); Kyoto Prefectural University of Medicine (S Hashimoto); Nara Medical University Hospital (H Fukushima)
*Malaysia*: Hospital Sultanah Nur Zahirah, Kuala Terengganu, Terengganu, (N Nik Adib); Kuala Lumpur Hospital (L Tai); Queen Elizabeth Hospital 2 (B Tony)
Philippines: Cebu Velez General Hospital (R Bigornia); Chong Hua Hospital (R Bigornia); Perpetual Succour Hospital (R Bigornia); The Medical City (J Palo)
*Singapore*: Alexandra Hospital (S Chatterjee); National University Health System (B Tan); Singapore General Hospital (A Kong); Tan Tock Seng Hospital (S Goh)
*Taiwan*: National Taiwan University Hospital (C Lee)
*Thailand*: Maharaj Nakorn Chiangmai Hospital, Chiaingmai University (C Pothirat); Prince of Songkla University (B Khwannimit); Ramathibodi Hospital (P Theerawit); Ramathibodi Hospital, Somdech Phra Debaratana Medical Centre (P Pornsuriyasak); Siriraj Hospital, Mahidol University (A Piriyapatsom)

***Middle East***
*Egypt*: Cairo University (A Mukhtar); Demerdash Surgical Intensive Care Unit (Dsicu); Ain Shams Faculty of Medicine (A Nabil Hamdy); Zaitoun Specialized Hospital (H Hosny)
*Iran*: Gums (A Ashraf); Imam Hossein Hospital, Sbums (M Mokhtari); Imamreza Hospital (S Nowruzinia); Laleh Hospital (A Lotfi); Shiraz University of Medical Sciences, SACRC (F Zand); Shiraz University of Medical Sciences (R Nikandish); Tehran Medical Sciences University (O Moradi Moghaddam)
*Israel*: Rabin Medical Centre (J Cohen); Sourasky Tel Aviv Medical Centre (O Sold)
*Lebanon*: Centre Hospitalier Du Nord (T Sfeir)
*Oman*: Sohar Hospital (A Hasan)
*Palestinian Territories*: Specialized Arab Hospital (D Abugaber)
*Saudi Arabia*: Almana General Hospital (H Ahmad); KFSHRC, Riyadh (T Tantawy); King Abdulaziz Medical City Riyadh (S Baharoom); King Abdulaziz University (H Algethamy); King Saud Medical City (A Amr); Riyadh Military Hospital (G Almekhlafi)
*Turkey*: Erciyes University Medical Faculty (R Coskun); Erciyes University Medical School (M Sungur); Gülhane Military Medical Academy (A Cosar); International Hospital, Istanbul (B Güçyetmez); Istanbul University Cerrahpasa Medical School Hospital (O Demirkiran); Istanbul University Istanbul Medical Faculty (E Senturk); Karadeniz Technical University, Medical Faculty (H Ulusoy); Memorial Atasehir Hospital (H Atalan); Pamukkale Universty (S Serin); Yuzuncu Yil Universitesy Medical Faculty (I Kati)
United Arab Emirates: Dubai Hospital (Z Alnassrawi); Mafraq Hospital (A Almemari); Sheikh Khalifa Medical City (K Krishnareddy); Tawam Hospital (S Kashef); The City Hospital (A Alsabbah)

***North America***
*Canada*: Hôpital Charles Lemoyne (G Poirier); St. Michael’s Hospital (J Marshall); Toronto General Hospital (M Herridge); Toronto Western Hospital (M Herridge)
*Puerto Rico*: San Juan Hospital (R Fernandez-Medero)
*United States*: Christiana Care Health System (G Fulda); Cincinnati Children's Hospital Medical Centre (S Banschbach); El Camino Hospital (J Quintero); George Washington Hospital (E Schroeder); Hospital of The University of Pennsylvania (C Sicoutris); John H Stroger Hospital of Cook County (R Gueret); Mayo Clinic, CCM (R Kashyap); Mayo Clinic, PCC (P Bauer); Medical College of Wisconsin (R Nanchal); Northwestern Memorial Hospital (R Wunderink); Orlando Regional Medical Centre (E Jimenez); Washington Hospital Centre (A Ryan); Washington Hospital Centre,2H (A Ryan); Washington Hospital Centre, 2G (A Ryan); Washington Hospital Centre, 3H (A Ryan); Washington Hospital Centre, 3G (A Ryan); Washington Hospital Centre, 4H (A Ryan); Washington Hospital Centre, CVRR (A Ryan)

***Oceania***
*Australia*: Armadale Health Service (D Prince); Bendigo Hospital (J Edington); Canberra Hospital (F Van Haren); Flinders Medical Centre (A Bersten); Joondalup Health Campus (DJ Hawkins); Lismore Base Hospital (M Kilminster); Mater Adult Hospital (D Sturgess); Prince Charles Hospital, Brisbane (M Ziegenfuss); Royal Adelaide Hospital (S O' Connor); Royal Brisbane and Womens’ Hospital (J Lipman); Royal Darwin Hospital (L Campbell); Royal Hobart Hospital (R Mcallister); Sir Charles Gairdner Hospital (B Roberts); The Queen Elizabeth Hospital (P Williams)
*New Zealand*: Auckland District Health Board (R Parke); Christchurch Hospital (P Seigne); Hawke's Bay Hospital (R Freebairn); Midcentral Health, Palmerston North Hospital (D Nistor); Middlemore Hospital (C Oxley); Wellington Hospital (P Young)

***South America***
*Argentina*: Cemic (Centro De Educación Médica E Investigaciones Clínicas) (R Valentini); Fleni (N Wainsztein); Hospital Aleman (P Comignani); Hospital Central San Isidro (M Casaretto); Hospital Fernandez (G Sutton); Hospital Francisco Lopez Lima Area Programa General Roca (P Villegas); Sanatorio Allende (C Galletti); Sanatorio De La Trinidad Palermo (J Neira); Sanatorio Julio Corzo Rosario (D Rovira)
*Belize*: Karl Heusner Memorial Hospital and Belize Healthcare Partner (J Hidalgo)
*Bolivia*: Hospital Obrero No1 (F Sandi)
*Brazil*: Cias -Unimed Vitória (E Caser); Evangelical Hospital of Cachoeiro De Itapemirim (M Thompson); Hospital 9 De Julho (M D'agostino Dias); Hospital Alcides Carneiro (L Fontes); Hospital Das Clínicas Luzia De Pinho Melo (M Lunardi); Hospital Das Nações De Curitiba (N Youssef); Hospital De Base Famerp (S Lobo); Hospital De Clínicas De Niterói (R Silva); Hospital De Clínicas Padre Miguel (J Sales Jr); Hospital De Terapia Intensiva (L Madeira Campos Melo); Hospital Do Trabalhador (M Oliveira); Hospital Esperanca (M Fonte); Hospital Evangelico De Londrina (C Grion); Hospital Geral De Fortaleza (C Feijo); Hospital Geral De Roraima (V Rezende); Hospital Israelita Albert Einstein (M Assuncao); Hospital Mater Dei (A Neves); Hospital Meridional (P Gusman); Hospital Meridional (D Dalcomune); Hospital Moinhos De Vento (C Teixeira); Hospital Municipal Ruth Cardoso (K Kaefer); Hospital Nereu Ramos (I Maia); Hospital Pasteur (V Souza Dantas); Hospital Pro Cardiaco (R Costa Filho); Hospital Regional De Samambaia (F Amorim); Hospital Regional Hans Dieter Schmidt (M Assef); Hospital Santa Casa - Campo Mourão (P Schiavetto); Hospital Santa Paula (J Houly); Hospital Santapaula (J Houly); Hospital São José Do Avaí (F Bianchi); Hospital São Lucas Da Pucrs (F Dias); Hospital Sao Vicente De Paula (C Avila); Hospital São Vicente De Paulo (J Gomez); Hospital Saude Da Mulher (L Rego); Hospital Tacchini (P Castro); Hospital Unimed Costa Do Sol-Macae-Rj (J Passos); Hospital Universitário - Ufpb - João Pessoa (C Mendes); Hospital Universitário De Londrina (C Grion); Hospital Universitário São Francisco (G Colozza Mecatti); Santa Casa De Caridade De Diamantina (M Ferrreira); Santa Casa De Misericordia De Tatui (V Irineu); São Francisco De Paula Hospital (M Guerreiro)
*Chile*: Clinica Indisa (S Ugarte); Clinica Las Lilas (V Tomicic); Hospital Carlos Van Buren (C Godoy); Hospital Del Trabajador De Santiago (W Samaniego); Hospital El Pino (I Escamilla); Hospital Mutual De Seguridad (I Escamilla)
*Colombia*: Centro Medico Imbanaco (L Castro Castro); Clinica Colombia Cali (G Libreros Duque); Clínica Del Café (D Diaz-Guio); Clínica La Estancia S.A. (F Benítez); Clinica Medellin (A Guerra Urrego); Fundacion Clinica Shaio (R Buitrago); Hospital Santa Clara (G Ortiz); Hospital Universitario Fundación Santa Fe De Bogota (M Villalba Gaviria)
Costa Rica: Calderón Guardia Hospital (D Salas); Hospital Dr Rafael Angel Varlderon Guardia Ccss (J Ramirez-Arce)
*Ecuador*: Clinica La Merced (E Salgado); Hospital Eugenio Espejo (D Morocho); Hospital Luis Vernaza (J Vergara); Shdug Sistema Hospitalario Docente De La Universidad De Guayaquil (M Chung Sang)
*El Salvador*: General Hospital (C Orellana-Jimenez)
*Guatemala*: Hospital Centro Medico (L Garrido)
*Honduras*: Instituto Hondureño Del Seguro Social (O Diaz)
*Martinique*: Centre Hospitalier Universitaire De Fort-De-France (D Resiere)
*Mexico*: Centro Estatal De Cuidados Críticos (C Osorio); Centro Médico Nacional "20 De Noviembre" Issste (A De La Vega); Fundacion Clinica Medica Sur (R Carrillo); Hospital San Jose TEC Monterrey (V Sanchez); Hospital 1o De Octubre, Issste (A Villagomez); Hospital Español De Mexico (R Martinez Zubieta); Hospital General Ajusco Medio (M Sandia); Hospital General Guadalupe Victoria (M Zalatiel); Hospital Juarez De Mexico (M Poblano); Hospitalcivil De Guadalajara, Hspitaljuan I Menchaca (D Rodriguez Gonzalez); Instituto Mexicano Del Seguro Social (F Arrazola); Instituto Mexicano Del Seguro Social (L Juan Francisco); Instituto Nacional de Cancerología, México (SA Ñamendys-Silva); ISSSTE Guerra Moya); Medical Centre ISSEMYM Toluca (M Hernandez); Mixta (D Rodriguez Cadena); Secretaria De Salud Del Distrito Federal (I Lopez Islas)
*Panama*: Hospital Santo Tomás (C Ballesteros Zarzavilla); Social Security Hospital (A Matos)
*Peru*: Clinica Anglo Americana (I Oyanguren); Essalud (J Cerna); Hospital Nacional Dos De Mayo (R Quispe Sierra); Hospital Rebagliati (R Jimenez); Instituto Nacional De Enfermedades Neoplasicas (L Castillo)
*Turks And Caicos Islands*: Gulhane Medical Faculty (R Ocal); Izmir Atatürk Educational And Research Hosp. (A Sencan)
*Uruguay*: CAMS (S Mareque Gianoni); CASMU (A Deicas); Hospital Español Asse (J Hurtado); Hospital Maciel (G Burghi)
*Venezuela*: Centro Medico De Caracas (A Martinelli); Hospital Miguel Perez Carreño (I Von Der Osten)

***South Asia***
*Afghanistan*: MSF Trauma Hospital Kunduz (C Du Maine)
*India*: Amri Hospitals (M Bhattacharyya); Amri Hospitals Salt Lake (S Bandyopadhyay); Apollo Hospital (S Yanamala); Apollo Hospitals (P Gopal); Apollo Hospitals, Bhubaneswar (S Sahu); Apollo Speciality Hospital (M Ibrahim); Asian Heart Institute (D Rathod); Baby Memorial Hospital Ltd, Calicut, Kerala (N Mukundan); Batra Hospital & Mrc, New Delhi 110062 (A Dewan); Bombay Hospital Institute of Medical Sciences (P Amin); Care Hospital (S Samavedam); Cims Hospital (B Shah); Columbiaasia Hospital, Mysore (D Gurupal); Dispur Hospitals (B Lahkar); Fortis Hospital (A Mandal); Fortis Hospital (Noida) (M Sircar); Fortis-Escorts Hospital, Faridabad, India (S Ghosh); Ganga Medical Centre & Hospital P Ltd. (V Balasubramani); Hinduja Hospital (F Kapadia); KDAH (S Vadi); Kerala Institute of Medical Sciences (KIMS, RMCC) (K Nair); Kalinga Institute of Medical Sciences (KIMS, DTEM) (S Tripathy); Kovai Medical Centre and Hospital (S Nandakumar); Medanta The Medicity, Gurgaon (J Sharma); Medica Superspecialty Hospitals (A Kar); Metro Heart Institute with Multispeciality (S Jha); Ruby Hall Pune (K Zirpe/Gurav); Saifee Hospital (M Patel); Spandan Multispeciality Hospital (A Bhavsar); Tata Main Hospital (D Samaddar); Tata Memorial Hospital (A Kulkarni)
*Pakistan*: Aga Khan University (M Hashmi); Hearts International Hospital (W Ali); Liaquat National Hospital (S Nadeem)
*Sri Lanka*: Sri Jayewardenepura General Hospital (K Indraratna)

***West Europe***
*Andorra*: Hospital Nostra Senyora De Meritxell (A Margarit)
*Austria*: Akh Wien (P Urbanek); Allgemeines Und Orthopädisches Landeskrankenhaus Stolzalpe (J Schlieber); Barmherzige Schwestern Linz (J Reisinger); General Hospital Braunau (J Auer); Krankenhaus D. Barmherzigen Schwestern Ried I.I. (A Hartjes); Krankenhaus Floridsdorf (A Lerche); LK Gmünd-Waidhofen/Thaya-Zwettl, Standort Zwettl (T Janous); LKH Hörgas-Enzenbach (E Kink); LKH West (W Krahulec); University Hospital (K Smolle)
*Belgium*: AZ Groeninge Kortrijk (M Van Der Schueren); AZ Jan Palfijn Gent (P Thibo); AZ Turnhout (M Vanhoof); Bracops Anderlecht (I Ahmet); Centre Hospitalier Mouscron (G Philippe); CH Peltzer La Tourelle (P Dufaye); Chirec Edith Cavell (O Jacobs); CHR Citadelle (V Fraipont); CHU Charleroi (P Biston); Chu Mont-Godinne (A Dive); CHU Tivoli (Y Bouckaert); Chwapi (E Gilbert); Clinique Saint-Pierre Ottignies (B Gressens); Clinique-Maternité Sainte Elisabeth (E Pinck); Cliniques De L'Europe - St-Michel (V Collin); Erasme University Hospital (JL Vincent); Ghent University Hospital (J De Waele); Moliere Hospital (R Rimachi); Notre Dame (D Gusu); Onze Lieve Vrouw Ziekenhuis, Aalst (K De Decker); Ixelles Hospital (K Mandianga); Sint-Augustinus (L Heytens); St Luc University Hospital (UCL) (X Wittebole); UZ Brussel (S Herbert); Vivalia Site De Libramont (V Olivier); VZW Gezondheidszorg Oostkust Knokke-Heist (W Vandenheede); ZNA Middelheim (P Rogiers)
*Denmark*: Herning Hospital (P Kolodzeike); Hjoerring Hospital (M Kruse); Vejle Hospital (T Andersen)
*Finland*: Helsinki University Central Hospital (V Harjola); Seinäjoki Central Hospital (K Saarinen)
*France*: Aix Marseille Univ, Hôpital Nord (M Leone); Calmette Hospital, Lille (A Durocher); Centre Hospitalier de Dunkerque (S Moulront); Centre Hospitalier Lyon Sud (A Lepape); Centre Hospitalo-Universitaire Nancy-Brabois (M Losser); CH Saint Philibert, Ghicl, Lille (P Cabaret); CHR De Dax (E Kalaitzis); CHU Amiens (E Zogheib); CHU Dijon (P Charve); CHU Dupuytren, Limoges (B François); CHU Nîmes (JY Lefrant); Centre Hospitalier De Troyes (B Beilouny); Groupe Hospitalier Est Francilien-Centre Hospitalier De Meaux (X Forceville); Groupe Hospitalier Paris Saint Joseph (B Misset); Hopital Antoine Béclère (F Jacobs); Hopital Edouard Herriot (F Bernard); Hôpital Lariboisère, APHP, Paris France (D Payen); Hopital Maison Blanche, Reims (A Wynckel); Hopitaux Universitaires de Strasbourg (V Castelain); Hospices Civils de Lyon (A Faure); CHU-Grenoble (P Lavagne); CHU-Nantes (L Thierry); Réanimation Chirurgical Cardiovasculaire, CHRU Lille (M Moussa); University Hospital Ambroise Paré (A Vieillard-Baron); University Hospital Grenoble (M Durand); University Hospital of Marseille (M Gainnier); University of Nice (C Ichai)
*Germany*: Alexianer Krefeld Gmbh (S Arens); Charite Hochschulmedizin Berlin (C Hoffmann); Charite-University-Hospital, Berlin (M Kaffarnik); Diakoniekrankenhaus Henriettenstiftung Gmbh (C Scharnofske); Elisabeth-Krankenhaus Essen (I Voigt); Harlaching Hospital, Munich Municipal Hospital Group (C Peckelsen); Helios St. Johannes Klinik (M Weber); Hospital St. Georg Leipzig (J Gille); Klinik Hennigsdorf Der Oberhavel Kliniken Gmbh (A Lange); Klinik Tettnang (G Schoser); Klinikum "St. Georg" Leipzig (A Sablotzki); Klinikum Augsburg (U Jaschinski); Klinikum Augsburg (A Bluethgen); Klinikum Bremen-Mitte (F Vogel); Klinikum Bremen-Ost (A Tscheu); Klinikum Heidenheim (T Fuchs); Klinikum Links Der Weser Gmbh (M Wattenberg); Klinikum Luedenscheid (T Helmes); Krankenhaus Neuwerk (S Scieszka); Marienkrankenhaus Schwerte (M Heintz); Medical Centre Cologne Merheim (S Sakka); Schwarzwald-Baar Klinikum Villingen-Schwenningen (J Kohler); St. Elisabeth Krankenhaus Köln-Hohenlind (F Fiedler); St. Martinus Hospital Olpe (M Danz); Uniklinikum Jena (Y Sakr); Universitätsklinikum Tübingen (R Riessen); Universitätsmedizin Mainz (T Kerz); University Hospital Aachen, CPACC (A Kersten); University Hospital Aachen, DMIII (F Tacke); University Hospital Aachen, OIC (G Marx); University Hospital Muenster (T Volkert); University Medical Centre Freiburg (A Schmutz); University Medical Centre Hamburg-Eppendorf (A Nierhaus); University Medical Centre Hamburg-Eppendorf (S Kluge); University Medicine Greifswald (P Abel); University of Duisburg-Essen (R Janosi); University of Freiburg (S Utzolino); University clinic Ulm (H Bracht); Vivantes Klinikum Neukoelln (S Toussaint)
*Greece*: Ahepa University Hospital (M Giannakou Peftoulidou); Athens University (P Myrianthefs); Athens University Medical School (A Armaganidis); Evangelismos Hospital (C Routsi); General Hospital of Chania, Crete (A Xini); Hippokration General Hospital, Thessaloniki (E Mouloudi); General hospital of Velos (I Kokoris); Lamia General Hospital (G Kyriazopoulos); Naval and Veterans Hospital (S Vlachos); Papanikolaou General Hospital (A Lavrentieva); University Hospital Alexandroupolis (P Partala); University of Ioannina (G Nakos)
*Iceland*: Landspitali University Hospital (A Moller); Landspitali University Hospital Fossvogur (S Stefansson)
*Ireland*: Cork University Hospital (J Barry); Mercy University Hospital (R O'Leary); Mid Western Regional Hospital Complex (C Motherway); Midland Regional Hospital Mullingar, Co Westmeath (M Faheem); St. Vincent's University Hospital (E Dunne); Tallaght Hospital (M Donnelly); University Hospital Galway (T Konrad)
*Italy*: Anesthesiology and Intensive Care (E Bonora); AO Ospedale Niguarda Ca' Granda (C Achilli); Azienda Ospedaliera Di Padova (S Rossi); Azienda Ospedaliero Universitaria Policlinico Vittorio Emanuele (G Castiglione); Careggi Teaching Hospital (A Peris); Clinicized Hospital Ss Annunziata - Chieti (D Albanese); Fondazione Irccs Ca' Granda Ospedale Maggiore Policlinico, Milano; University of Milan (N Stocchetti); H San Gerardo - Monza (G Citerio); Icu "Ceccarini" Hospital Riccione (L Mozzoni); Irccs Centro Cardiologico Monzino (E Sisillo); Irccs Centro Di Riferimento Oncologico Della Basilicata (P De Negri); Irccs Fondazione Ca' Granda - Ospedale Maggiore Policlinico (M Savioli); Ospedale Belcolle Viterbo (P Vecchiarelli); Ospedale Civile Maggiore - A.O.U.I Verona (F Puflea); Ospedale Civile Maggiore - A.O.U.I Verona (V Stankovic); Ospedale Di Circolo E Fondazione Macchi - Varese (G Minoja); Ospedale Di Trento - Azienda Provinciale Per I Servizi Sanitari Della Provincia Autonoma Di Trento (S Montibeller); Ospedale Orlandi (P Calligaro); Ospedale Regionale U.Parini-Aosta (R Sorrentino); Ospedale San Donato Arezzo (M Feri); Ospedale San Raffaele (M Zambon); Policlinico G.B. Rossi - A.O.U.I Verona (E Colombaroli); Policlinico University of Palermo (A Giarratano); Santa Maria Degli Angeli Hospital (T Pellis); Saronno Hospital (C Capra); Università Cattolica Del Sacro Cuore (M Antonelli); University Catania, Italy (A Gullo); University of Florence, Florence (C Chelazzi); University of Foggia (A De Capraris); University of Milano-Bicocca, San Gerardo Hospital (N Patroniti); University of Modena (M Girardis); University of Siena (F Franchi); University of Trieste (G Berlot)
*Malta*: Mater Dei Hospital (M Buttigieg)
*Netherlands*: Albert Schweitzer Hospital (H Ponssen); Antoni Van Leeuwenhoek Ziekenhuis (J Ten Cate); Atrium Medisch Centrum Parkstad (L Bormans); Bovenij Hospital (S Husada); Catharina Hospital Eindhoven (M Buise); Erasmus University Medical Centre (B Van Der Hoven); Martiniziekenhuis Groningen (A Reidinga); Medical Centre Leeuwarden (M Kuiper); Radboud University Nijmegen Medical Centre (P Pickkers); Slotervaart Ziekenhuis Amsterdam (G Kluge); Spaarne Ziekenhuis (S Den Boer); University Medical Centre Utrecht (J Kesecioglu); Ziekenhuis Rijnstate (H Van Leeuwen)
*Norway*: Haukeland University Hospital (H Flaatten); St Olavs Hospital, Trondheim University Hospital (S Mo)
*Portugal*: Centro Hospitalar Cova Da Beira (V Branco); Centro Hospitalar Do Porto (F Rua); Centro Hospitalar Do Tâmega E Sousa (E Lafuente); Centro Hospitalar Gaia/Espinho, Epe (M Sousa); Centro Hospitalar Médio Tejo (N Catorze); Centro Hospitalar Tondela-Viseu (M Barros); Faro Hospital (L Pereira); Hospital Curry Cabral (A Vintém De Oliveira); Hospital Da Luz (J Gomes); Hospital De Egas Moniz - Chlo (I Gaspar); Hospital De Santo António, Centro Hospitalar Do Porto (M Pereira); Hospital Divino Espírito Santo, Epe (M Cymbron); Hospital Espirito Santo - Évora Epe (A Dias); Hospital Garcia Orta (E Almeida); Hospital Geral Centro Hospitalar E Universitario Coimbra (S Beirao); Hospital Prof. Doutor Fernando Fonseca Epe (I Serra); Hospital São Bernardo (R Ribeiro); Hospital Sao Francisco Xavier, Chlo (P Povoa); Instituto Portugues De Oncologia Francisco Gentil, Porto (F Faria); Santa Maria Hospital (Z Costa-E-Silva); Serviço De Saúde Da Região Autonóma Da Madeira (J Nóbrega); UCIP (F Fernandes); ULS - Castelo Branco (J Gabriel)
*Slovenia*: General Hospital Celje (G Voga); General Hospital Izola (E Rupnik); General Hospital Novo Mesto (L Kosec); Oncological Institute (M Kerin Povšic); Ukc Maribor (I Osojnik); University Clinic of Respiratory and Allergic Diseases (V Tomic); University Clinical Centre Maribor (A Sinkovic)
*Spain*: CH Salamanca (J González); Clinic Hospital (E Zavala); Complejo Hospitalario De Jaén (J Pérez Valenzuela); Complejo Hospitalario De Toledo (L Marina); Complexo Hospitalario Universitario De Ourense (P Vidal-Cortés); Complexo Hospitalario Universitario De Vigo (P Posada); Corporación Sanitaria Parc Tauli (A Ignacio Martin-Loeches); Cruz Roja Hospital (N Muñoz Guillén); H Vall Hebron (M Palomar); HGGC Dr Negrín (J Sole-Violan); Hospital Clinic (A Torres); Hospital Clinico San Carlos (M Gonzalez Gallego); Hospital Clínico Universitario De Valencia (G Aguilar); Hospital Clínico Universitario Lozano Blesa (R Montoiro Allué); Hospital Clinico Valencia (M Argüeso); Hospital De La Ribera (M Parejo); Hospital De Sagunto (M Palomo Navarro); Hospital De San Juan De Alicante (A Jose); Hospital De Torrejon De Ardoz (N Nin); Hospital Del Mar (F Alvarez Lerma); Hospital Del Tajo (O Martinez); Hospital General Universitario De Elche (E Tenza Lozano); Hospital General Universitario Gregorio Marañon (S Arenal López); Hospital General Universitario Gregorio Marañon (M Perez Granda); Hospital General Universitario Santa Lucía (S Moreno); Hospital Germans Trias I Pujol (C Llubia); Hospital Infanta Margarita (C De La Fuente Martos); Hospital Infanta Sofia (P Gonzalez-Arenas); Hospital J.M. Morales Meseguer (N Llamas Fernández); Hospital J.M. Morales Meseguer (B Gil Rueda ); Hospital Marina Salu. Denia. Alicante. (I Estruch Pons); Hospital Nuestra Señora Del Prado, Talavera De La Reina, Toledo. España (N Cruza); Hospital San Juan De Dios Aljarafe (F Maroto); Hospital Sas of Jerez (A Estella); Hospital Son Llatzer (A Ferrer); Hospital Universitario Central De Asturias (L Iglesias Fraile); Hospital Universitario Central De Asturias (B Quindos); Hospital Universitario De Alava, Santiago (A Quintano); Hospital Universitario De Basurto, Bilbao (M Tebar); Hospital Universitario de Getafe (P Cardinal); Hospital Universitario De La Princesa (A Reyes); Hospital Universitario de Tarragona Joan Xxiii (A Rodríguez); Hospital Universitario Del Henares (A Abella); Hospital Universitario Fundación Alcorcón (S García Del Valle); Hospital Universitario La Paz (S Yus); Hospital Universitario La Paz (E Maseda); Hospital Universitario Rio Hortega (J Berezo); Hospital Universitario San Cecilio (Granada) (A Tejero Pedregosa); Hospital Virgen Del Camino (C Laplaza); Mutua Terrassa University Hospital (R Ferrer); Rã­o Hortega University Hospital (J Rico-Feijoo); Servicio Andaluz De Salud. Spain. (M Rodríguez); University okalingaf Navarra (P Monedero)
*Sweden*: Karolinska University Hospital And Karolinska Institute (K Eriksson); Sunderby Hospital, Luleå (D Lind)
*Switzerland*: Hôpital Intercantonal De La Broye (D Chabanel); Hôpital Neuchâtelois - La Chaux-De-Fonds (H Zender); Lindenhofspital (K Heer); Regionalspital Surselva Ilanz (Gr) Schweiz (B Frankenberger); University Hospital Bern (S Jakob); Zentrum Für Intensivmedizin (A Haller)
*United Kingdom*: Alexandra Hospital Redditch (S Mathew); Blackpool Teaching Hospitals (R Downes); Brighton And Sussex University Hospitals (C Barrera Groba); Cambridge University Hospitals NHS Foundation Trust (A Johnston); Charing Cross Hospital (R Meacher); Chelsea & Westminster Hospital (R Keays); Christie Foundation Trust (P Haji-Michael); County Hospital, Lincoln (C Tyler); Craigavon Area Hospital (A Ferguson); Cumberland Infirmary (S Jones); Darent Valley Hospital (D Tyl); Dorset County Hospital (A Ball); Ealing Hospital NHS Trust (J Vogel); Glasgow Royal Infirmary (M Booth); Gloucester Royal Hospital (P Downie); The Great Western Hospital, Swindon (M Watters); Imperial College Healthcare NHS Trust (S Brett); Ipswich Hospital Nhs Trust (M Garfield); James Paget University Hospital NHS Foundation Trust (L Everett); King's College Hospital (S Heenen); King's Mill Hospital (S Dhir); Leeds Teaching Hospitals NHS Trust (Z Beardow); Lewisham Healthcare NHS Trust (M Mostert); Luton and Dunstable Hospital NHS Trust (S Brosnan); Medway Maritime Hospital (N Pinto); Musgrove Park Hospital (S Harris); Nevill Hall Hospital (A Summors); Pilgrim Hospital (N Andrew); Pinderfields Hospital, Mid Yorkshire NHS Trust (A Rose); Plymouth Hospitals Nhs Trust (R Appelboam); Princess Royal Hospital Telford (O Davies); Royal Bournemouth Hospital (E Vickers); Royal Free Hampstead NHS Foundation Trust (B Agarwal); Royal Glamorgan Hospital (T Szakmany); Royal Hampshire County Hospital (S Wimbush); Royal Liverpool University Hospital (I Welters); Royal London Hospital, Barts Health NHS Trust (R Pearse); Royal Shrewsbury Hospital (R Hollands); Royal Surrey County Hospital (J Kirk-Bayley); St Georges Healthcare (N Fletcher); Surrey & Sussex Healthcare Trust (B Bray); University College Hospital (D Brealey)
