# Supplementary material for: A worldwide multicentre evaluation of the influence of deterioration or improvement of acute kidney injury on clinical outcome in critically ill patients with and without sepsis at ICU admission: results from The Intensive Care Over Nations audit
Source: Crit Care. 2018 Aug 3;22:188. doi: 10.1186/s13054-018-2112-z (PMC6091052; doi:10.1186/s13054-018-2112-z)
Supplement: Supplementary file 3 — Table S1. Adjusted AKIN criteria for diagnosis of AKI. Table S2. RRT modality per day. Table S3. RRT and mortality per region and GNI. Table S4. RRT modality per region and GNI. Table S5. Length of stay per region and GNI. Table S6. Mortality per RRT modality. Table S7. Baseline demographic characteristics of patients with CRF, with and without sepsis. Table S8. Baseline clinical characteristics of patients with CRF, with and without sepsis. Table S9. Outcome of patients with CRF at admission (DOCX 48 kb) [file 13054_2018_2112_MOESM3_ESM.docx]

**Table S1. Adjusted AKIN criteria for diagnosis of acute kidney injury**

|  | **Serum creatinine** | **Original urine output criteria** | **Adjusted urine output criteria** |
| --- | --- | --- | --- |
| Stage 1 | Abrupt increase in creatinine x 1.5-2 within 48 hours | <0.5 ml/kg/h x 6h | <1.0 ml/kg/h x 24h |
| Stage 2 | Increase creatinine x 2-3 | <0.5 ml/kg/h x 12h | <0.5 ml/kg/h x 24h |
| Stage 3 | Increase creatinine x >3 or creatinine ≥ 4 mg/100 ml  (acute rise of ≥ 0.5 mg/100 ml) | <0.3 ml/kg/h x 24h  or anuria x 12h | <0.3 ml/kg/h x 24h  or anuria x 24h |

ESRD: end-stage renal disease.

**Table S2. Renal replacement therapy modality per day**

|  | **Total population** | |  | **Sepsis** | |  | **No sepsis** | |
| --- | --- | --- | --- | --- | --- | --- | --- | --- |
|  | *Haemodialysis* | *Haemofiltration* |  | *Haemodialysis* | *Haemofiltration* |  | *Haemodialysis* | *Haemofiltration* |
| 0 | 325 (66) | 278 (56) |  | 143 (61) | 152 (65)* |  | 182 (70) | 126 (48) |
| 1 | 352 (61) | 371 (64) |  | 178 (56)* | 224 (71)* |  | 174 (66) | 147 (56) |
| 2 | 265 (58) | 302 (66) |  | 143 (54)* | 192 (72)* |  | 122 (65) | 110 (58) |
| 3 | 229 (61) | 248 (66) |  | 130 (57)* | 159 (69) |  | 99 (68) | 89 (61) |
| 4 | 175 (61) | 184 (65) |  | 112 (57)* | 134 (69)* |  | 63 (70) | 50 (56) |
| 5 | 142 (62) | 142 (62) |  | 94 (59) | 100 (63) |  | 48 (68) | 42 (59) |
| 6 | 119 (60) | 127 (64) |  | 81 (58) | 91 (65) |  | 38 (66) | 36 (62) |
| 7 | 91 (57) | 104 (65) |  | 69 (58) | 74 (62) |  | 22 (56) | 30 (77) |

Data presented as number (%). Day 0 is day of ICU admission. Significant at 5% level: * vs. No sepsis patients with the same treatment modality.

**Table S3. Renal replacement therapy (RRT) and mortality per region and gross national income (GNI)**

|  |  |  | **Sepsis** | | | | |  | **No sepsis** | | | | |
| --- | --- | --- | --- | --- | --- | --- | --- | --- | --- | --- | --- | --- | --- |
|  |  |  | *All* | *No AKI* | *Stage 1* | *Stage 2* | *Stage 3* |  | *All* | *No AKI* | *Stage 1* | *Stage 2* | *Stage 3* |
| RRT, | Region | Africa | 4 (20.0) | 1 (25) | 0 (0)‡ | (0)‡ | 3 (33.3) |  | 16 (19.0)‡ | 2 (7.4) | 2 (7.1) | 1 (11.1) | 11 (55)# |
| n (%) |  | Middle East | 23 (22.8)$ | 2 (5.4) | 6 (19.4)‡ | 0 (0)‡ | 15 (55.6)# |  | 19 (9.8)‡ | 2 (2.1) | 4 (6.9) | 1 (5.6) | 12 (52.2)# |
|  |  | South America | 46 (23.1)$ | 1 (2.6) | 5 (8.6)* | 3 (9.4) | 37 (52.1)#* |  | 31 (5.6) | 3 (1.3) | 1 (0.5) | 1 (1.7) | 26 (31.0)# |
|  |  | North America | 10 (11.2)$‡ | 2 (6.5) | 0 (0)‡ | 0 (0)‡ | 8 (33.3)#‡ |  | 13 (2.6) | 0 (0)‡ | 1 (0.7) | 1 (1.8) | 11 (15.9)#‡ |
|  |  | West Europe | 176 (20.1)$ | 7 (2.6) | 13 (5.0)* | 11 (10.1)#* | 145 (61.2)#* |  | 134 (5.4) | 17 (1.6) | 7 (0.8) | 3 (1.2) | 107 (35.3)# |
|  |  | East Europe | 38 (18.2)$ | 5 (6.0)* | 1 (2.1) | 0 (0)#‡ | 32 (54.2)#* |  | 28 (4.0) | 1 (0.3)‡ | 3 (1.2) | 0 (0) | 24 (27.9)# |
|  |  | Oceania | 15 (15.6)$ | 1 (2.9) | 1 (2.9) | 0 (0)‡ | 13 (86.7)#*‡ |  | 9 (3.3) | 0 (0)‡ | 1 (1.2) | 1 (2.3) | 7 (20.6)#‡ |
|  |  | East and southeast Asia | 51 (18.9)$ | 6 (6.1)* | 6 (7.0)* | 1 (3.7) | 38 (65.5)# |  | 50 (10.3)‡ | 0 (0)‡ | 1 (0.8) | 0 (0) | 49 (53.3)#‡ |
|  |  | South Asia | 17 (19.5)$ | 0 (0)‡ | 1 (3.7) | 0 (0)‡ | 16 (66.7)#* |  | 26 (3.4) | 2 (0.7) | 2 (0.8) | 2 (1.9) | 20 (21.1)#‡ |
|  | GNI | Low and lower-middle income | 19 (14.6)$ | 1 (2.2) | 1 (2.6) | 0 (0)‡ | 17 (43.6)#*‡ |  | 30 (3.3) | 3 (0.8) | 2 (0.7) | 2 (1.6) | 23 (19.7)#‡ |
|  |  | Upper-middle income | 110 (20.6)$ | 9 (5.3)* | 14 (8.8)* | 4 (6.7) | 83 (57.2)#* |  | 112 (7.7) | 6 (1.0) | 7 (1.5) | 2 (1.5) | 97 (43.5)#‡ |
|  |  | High income | 251 (19.6)$ | 15 (3.6)* | 18 (4.8)* | 11 (7.2)* | 207 (60.9)#* |  | 184 (5.0) | 18 (1.1) | 13 (1.1) | 6 (1.6) | 147 (31.5)# |
|  |  |  |  |  |  |  |  |  |  |  |  |  |  |
| ICU mortality, | Region | Africa | 6 (30.0)$ | 0 (0)‡ | 0 (0)‡ | 0 (0)‡ | 6 (66.7)#* |  | 6 (7.4) | 2 (8.0) | 1 (3.7) | 0 (0)‡ | 3 (15)‡ |
| n (%) |  | Middle East | 35 (35.4)$ | 7 (18.9) | 10 (33.3) | 0 (0)*‡ | 18 (66.7)#‡ |  | 35 (18.6)‡ | 10 (11.0) | 11 (19.3)‡ | 4 (22.2) | 10 (45.5)# |
|  |  | South America | 78 (41.9)$‡ | 8 (24.2) | 18 (32.7)*‡ | 12 (42.9)‡ | 40 (57.1)#*‡ |  | 73 (14.0) | 27 (12.6) | 14 (8.1) | 13 (22.4) | 19 (24.4)#‡ |
|  |  | North America | 17 (19.1)$ | 4 (12.9) | 5 (20.0)* | 1 (11.1) | 7 (29.2) |  | 33 (6.6)‡ | 14 (6.0) | 5 (3.4)‡ | 5 (9.1) | 9 (13.2)‡ |
|  |  | West Europe | 179 (20.6)$ | 35 (14.2)* | 43 (16.5)* | 16 (14.7) | 85 (36.2)# |  | 279 (11.5) | 89 (8.4) | 57 (6.9) | 27 (11.2) | 106 (35.6)# |
|  |  | East Europe | 76 (36.4)$‡ | 27 (32.5)*‡ | 11 (23.4)* | 11 (55.0)*‡ | 27 (45.8) |  | 101 (14.5) | 38 (12.1) | 24 (10.0) | 9 (16.1) | 30 (34.9)# |
|  |  | Oceania | 11 (11.5) | 2 (5.7) | 4 (11.4) | 1 (9.1) | 4 (26.7) |  | 23 (8.4) | 5 (4.4) | 7 (8.3) | 4 (9.3) | 7 (20.6)#‡ |
|  |  | East and southeast Asia | 61 (23.3)$ | 18 (19.1)* | 19 (22.6)* | 6 (22.2) | 18 (31.6) |  | 58 (12.3) | 17 (7.9) | 8 (6.2) | 7 (17.9) | 26 (29.2)# |
|  |  | South Asia | 28 (33.3)$ | 7 (21.9)* | 7 (28.0)* | 3 (100)*‡ | 11 (45.8)* |  | 47 (6.8)‡ | 11 (4.0)‡ | 11 (4.5) | 7 (7.5) | 18 (20.7)#‡ |
|  | GNI | Low and lower-middle income | 46 (36.5)$‡ | 13 (28.9)*‡ | 10 (27.8)* | 5 (83.3)*‡ | 18 (46.2)* |  | 82 (9.8) | 21 (6.3) | 19 (6.7) | 14 (12.3) | 28 (25.7)# |
|  |  | Upper-middle income | 179 (34.9)$‡ | 42 (26.1)*‡ | 47 (30.5)*‡ | 21 (38.2)*‡ | 69 (48.3)#*‡ |  | 189 (13.4)‡ | 65 (10.8)‡ | 43 (9.3) | 22 (16.9) | 59 (27.7)# |
|  |  | High income | 266 (20.9)$ | 53 (13.0)* | 60 (15.9)* | 24 (15.8) | 129 (38.2)#* |  | 239 (18.1) | 127 (8.0) | 76 (6.4) | 40 (10.9) | 141 (30.7)# |
|  |  |  |  |  |  |  |  |  |  |  |  |  |  |
| Hospital mortality, | Region | Africa | 7 (38.9)$ | 1 (33.3) | 0 (0)‡ | 0 (0)‡ | 6 (66.7)* |  | 6 (8.3) | 2 (9.5) | 1 (4.2)‡ | 0 (0)‡ | 3 (15)‡ |
| n (%) |  | Middle East | 45 (45.9)$ | 11 (30.6)* | 13 (43.3) | 1 (20.0) | 20 (74.1)#‡ |  | 42 (23.5)‡ | 11 (12.8) | 15 (27.3)#‡ | 5 (29.4) | 11 (52.4)# |
|  |  | South America | 88 (50.6)$‡ | 9 (30.0) | 21 (40.4)*‡ | 13 (50.0) | 45 (68.2)#*‡ |  | 98 (20.4) | 33 (16.9) | 24 (15.5) | 16 (28.6) | 25 (33.8)# |
|  |  | North America | 22 (24.7)$ | 5 (16.1) | 7 (28.0) | 2 (22.2) | 8 (33.3) |  | 48 (9.6)‡ | 17 (7.3)‡ | 11 (7.5)‡ | 7 (12.7) | 13 (19.4)#‡ |
|  |  | West Europe | 267 (31.5)$ | 59 (22.8)* | 64 (25.4)* | 34 (31.8)* | 110 (48.0)# |  | 394 (16.7) | 130 (12.6) | 102 (12.8) | 43 (18)# | 119 (40.6)# |
|  |  | East Europe | 90 (44.3)$‡ | 32 (40.0)*‡ | 16 (34.0)* | 11 (57.9)*‡ | 31 (54.4) |  | 119 (17.6) | 46 (15.0) | 29 (12.4) | 10 (18.5) | 34 (41.0)# |
|  |  | Oceania | 18 (18.8)$‡ | 4 (11.4) | 6 (17.1) | 3 (27.3) | 5 (33.3) |  | 28 (10.3)‡ | 5 (4.4)‡ | 10 (12.2) | 4 (9.3) | 9 (26.5)# |
|  |  | East and southeast Asia | 81 (32.0)$ | 23 (25.6)* | 28 (34.1)* | 7 (25.9) | 23 (42.6)# |  | 75 (16.9) | 24 (11.7) | 11 (9.4) | 10 (26.3) | 30 (35.7)# |
|  |  | South Asia | 33 (39.8)$ | 10 (32.3)* | 7 (28.0)* | 3 (100)#*‡ | 13 (54.2)* |  | 59 (9.3)‡ | 18 (7.3)‡ | 16 (7.0)‡ | 7 (9.0)‡ | 18 (22.2) #‡ |
|  | GNI | Low and lower-middle income | 53 (42.7)$‡ | 16 (36.4)* | 10 (27.8)* | 5 (83.3)#*‡ | 22 (57.9)#* |  | 98 (12.7) | 31 (10.2) | 25 (9.4) | 14 (14.4) | 28 (27.2)# |
|  |  | Upper-middle income | 207 (42.4)$‡ | 49 (32.5)*‡ | 58 (39.2)*‡ | 23 (43.4)* | 77 (56.6)#* |  | 239 (18.1)‡ | 81 (14.3) | 60 (14.1) | 29 (23.0)# | 69 (34.0)# |
|  |  | High income | 391 (31.3)$ | 89 (22.3)* | 94 (25.5)* | 46 (30.9)#* | 162 (48.9)#* |  | 532 (15.1) | 174 (11.1) | 134 (11.7) | 59 (16.2)# | 165 (36.6)# |

AKI: acute kidney injury; Significant at 5% level: # vs. No AKI in the same group (sepsis or no sepsis), * vs. No sepsis patients with the same AKIN stage, ‡ vs. West Europe or High income with the same AKIN stage or the total population, in the same group (sepsis or no sepsis), $ vs. all patients without sepsis.

**Table S4. Renal replacement therapy modality per region and gross national income (GNI)**

|  | **Total population** | |  | **Sepsis** | |  | **No sepsis** | |
| --- | --- | --- | --- | --- | --- | --- | --- | --- |
|  | *Haemodialysis* | *Haemodialysis* |  | *Haemodialysis* | *Haemofiltration* |  | *Haemodialysis* | *Haemofiltration* |
| Region |  |  |  |  |  |  |  |  |
| Africa | 14 (19.2) | 5 (6.8) |  | 2 (12.5) | 2 (12.5) |  | 12 (21.1) | 3 (5.3) |
| Middle East | 27 (16.6) | 25 (15.3) |  | 14 (21.9) | 17 (26.6)* |  | 13 (13.1) | 8 (8.1) |
| South America | 66 (13.5) | 45 (9.2) |  | 42 (26.1)* | 28 (17.4)* |  | 24 (7.3) | 17 (5.2) |
| North America | 17 (5.2) | 10 (3.0) |  | 6 (10.3) | 5 (8.6)* |  | 11 (4.1) | 5 (1.9) |
| West Europe | 155 (7.8) | 221 (11.1) |  | 90 (14.9)* | 141 (23.3)* |  | 65 (4.7) | 80 (5.8) |
| East Europe | 41 (8.0) | 34 (6.7) |  | 22 (17.5)* | 22 (17.5)* |  | 19 (4.9) | 12 (3.1) |
| Oceania | 11 (5.0) | 21 (9.5) |  | 8 (13.1)* | 13 (21.3)* |  | 3 (1.9) | 8 (5.0) |
| East and southeast Asia | 50 (11.5) | 71 (16.3) |  | 25 (14.6) | 38 (22.2)* |  | 25 (9.5) | 33 (12.5) |
| South Asia | 34 (6.6) | 18 (3.5) |  | 14 (25.5)* | 7 (12.7)* |  | 20 (4.4) | 11 (2.4) |
| GNI |  |  |  |  |  |  |  |  |
| Low and lower-middle income | 38 (6.1) | 21 (3.4) |  | 15 (17.9)* | 8 (9.5)* |  | 23 (4.2) | 13 (2.4) |
| Upper-middle income | 148 (12.4) | 126 (10.5) |  | 78 (21.4)* | 68 (18.6)* |  | 70 (8.4) | 58 (7.0) |
| High income | 229 (7.9) | 303 (10.4) |  | 130 (15.0)* | 197 (22.7)* |  | 99 (4.9) | 106 (5.2) |

Data presented as number (%). Significant at 5% level: * vs. patients without sepsis with the same treatment modality.

**Table S5. Length of stay per region and gross national income (GNI)**

|  |  |  | **Sepsis** | | | | |  | **No sepsis** | | | | |
| --- | --- | --- | --- | --- | --- | --- | --- | --- | --- | --- | --- | --- | --- |
|  |  |  | *All* | *No AKI* | *Stage 1* | *Stage 2* | *Stage 3* |  | *All* | *No AKI* | *Stage 1* | *Stage 2* | *Stage 3* |
| ICU LOS, | Region | Africa | 13 [6-60]$‡ | 13 [8-24]* | 6 [4-6]#*‡ | 15 [15-15]* | 60 [10-60]#*‡ |  | 2 [1-4]‡ | 2 [1-4] | 2 [1-3]#‡ | 3 [3-5]#‡ | 2 [2-7]#‡ |
| days |  | Middle East | 13 [6-60]$‡ | 9 [6-27]*‡ | 15 [8-60]#*‡ | 5 [4-5]#*‡ | 60 [11-60]#*‡ |  | 3 [2-14]‡ | 3 [2-6]‡ | 3 [2-8]#‡ | 7 [2-32]#‡ | 6 [3-60]#‡ |
|  |  | South America | 21 [7-60]$‡ | 11 [5-60]*‡ | 18 [6-60]#*‡ | 14 [5-60]#*‡ | 60 [14-60]#*‡ |  | 4 [2-7]‡ | 4 [2-7]‡ | 4 [2-7]#‡ | 3 [2-14]#‡ | 5 [3-46]#‡ |
|  |  | North America | 3 [2-16]$‡ | 2 [1-14]*‡ | 3 [2-16]#* | 2 [2-4]#‡ | 6 [2-60]#*‡ |  | 2 [1-4]‡ | 2 [1-4]‡ | 2 [1-3]#‡ | 2 [1-4]#‡ | 2 [1-7]#‡ |
|  |  | West Europe | 7 [3-29]$ | 6 [2-15]* | 6 [3-18]#* | 8 [3-21]#* | 15 [5-60]#* |  | 3 [1-6] | 2 [1-5] | 2 [1-5]# | 2 [1-6]# | 5 [2-60]# |
|  |  | East Europe | 13 [4-60]$‡ | 13 [5-60]*‡ | 7 [3-60]#*‡ | 60 [5-60]#*‡ | 15 [5-60]#*‡ |  | 3 [2-7]‡ | 3 [2-6]‡ | 3 [2-5]#‡ | 3 [1-7]#‡ | 6 [2-60]# |
|  |  | Oceania | 4 [2-10]$‡ | 3 [2-6]*‡ | 4 [2-9]#*‡ | 4 [2-9]#* | 11 [4-60]#*‡ |  | 2 [1-4]‡ | 2 [1-3]‡ | 2 [1-5]# | 2 [1-6]#‡ | 3 [2-12]#‡ |
|  |  | East and southeast Asia | 9 [5-41]$‡ | 8 [4-26]*‡ | 8 [5-35]*‡ | 8 [4-16]* | 12 [6-60]#*‡ |  | 3 [2-7]‡ | 3 [2-6] | 3 [2-7]# | 3 [2-8]#‡ | 6 [3-60]#‡ |
|  |  | South Asia | 7 [3-60]$‡ | 6 [3-29]*‡ | 5 [3-60]*‡ | 60 [60-60]#*‡ | 20 [5-60]#* |  | 2 [1-4]‡ | 2 [1-4]‡ | 3 [1-4]#‡ | 2 [1-4]#‡ | 2 [1-6]#‡ |
|  | GNI | Low and lower-middle income | 10 [4-60]$‡ | 8 [4-60]*‡ | 6 [3-60]*‡ | 60 [60-60]#*‡ | 21 [6-60]#*‡ |  | 3 [1-5]‡ | 3 [1-4]‡ | 3 [2-5]# | 2 [1-5]# | 3 [1-60]#‡ |
|  |  | Upper-middle income | 12 [5-60]$‡ | 9 [5-60]*‡ | 10 [5-60]#*‡ | 10 [5-60]#*‡ | 29 [7-60]#*‡ |  | 3 [2-7]‡ | 3 [2-6]‡ | 3 [2-7]#‡ | 3 [2-8]#‡ | 5 [2-60]#‡ |
|  |  | High income | 7 [3-30]$ | 6 [2-15]* | 6 [3-18]# | 7 [3-21]#* | 16 [5-60]#* |  | 2 [1-5] | 2 [1-5] | 2 [1-5]# | 2 [1-6]# | 4 [2-60]# |
|  |  |  |  |  |  |  |  |  |  |  |  |  |  |
| Hospital LOS, | Region | Africa | 38 [21-60]$ | 60 [44-60]*‡ | 19 [9-24]#*‡ | 32 [32-32]#*‡ | 60 [23-60]* |  | 9 [4-14]‡ | 10 [5-13]‡ | 5 [3-15]#‡ | 7 [4-25]‡ | 9 [3-15]‡ |
| days |  | Middle East | 60 [22-60]$ | 46 [19-60]*‡ | 60 [24-60]#*‡ | 11 [10-19]#*‡ | 60 [60-60]#*‡ |  | 14 [7-60]‡ | 14 [7-29]‡ | 13 [7-60]#‡ | 39 [11-60]#‡ | 60 [7-60]#‡ |
|  |  | South America | 60 [24-60]$‡ | 55 [20-60]*‡ | 51 [21-60]*‡ | 60 [25-60]#*‡ | 60 [60-60]#*‡ |  | 12 [6-60]‡ | 12 [6-36]‡ | 11 [6-36]‡ | 12 [6-60]‡ | 20 [6-60]#‡ |
|  |  | North America | 20 [7-60]$‡ | 19 [7-50]*‡ | 15 [6-60]*‡ | 7 [5-12]#*‡ | 27 [11-60]#*‡ |  | 7 [4-15]‡ | 7 [4-14]‡ | 6 [4-14]‡ | 8 [4-16]#‡ | 9 [4-52]#‡ |
|  |  | West Europe | 36 [15-60]$ | 24 [14-60]* | 29 [15-60]#* | 50 [17-60]#* | 60 [22-60]#* |  | 13 [7-34] | 11 [7-25] | 13 [7-29]# | 13 [7-34]# | 30 [11-60]# |
|  |  | East Europe | 59 [14-60]$‡ | 33 [13-60]*‡ | 31 [10-60]* | 60 [16-60]#*‡ | 60 [19-60]#* |  | 13 [8-41]‡ | 12 [7-25]‡ | 13 [8-22] | 14 [9-60]#‡ | 43 [8-60]# |
|  |  | Oceania | 19 [8-60]$‡ | 14 [7-32]*‡ | 21 [8-59]#*‡ | 24 [5-60]#*‡ | 51 [12-60]#* |  | 9 [5-18]‡ | 7 [4-15]‡ | 8 [5-16]#‡ | 11 [6-22]#‡ | 15 [6-60]#‡ |
|  |  | East and southeast Asia | 32 [12-60]$‡ | 22 [11-60]* | 30 [10-60]#* | 25 [14-60]‡ | 60 [19-60]#*‡ |  | 15 [7-52]‡ | 14 [7-35]‡ | 12 [6-30]# | 20 [6-60]#‡ | 38 [8-60]# |
|  |  | South Asia | 19 [7-60]$‡ | 16 [9-60]*‡ | 10 [5-60]#*‡ | 60 [60-60]#*‡ | 60 [14-60]#*‡ |  | 6 [2-11]‡ | 7 [3-11]‡ | 7 [3-10]#‡ | 5 [2-9]‡ | 6 [2-28]#‡ |
|  | GNI | Low and lower-middle income | 29 [10-60]$ | 20 [9-60]*‡ | 13 [7-60]#*‡ | 60 [60-60]#*‡ | 60 [20-60]#* |  | 7 [3-13]‡ | 7 [3-14]‡ | 7 [3-11]#‡ | 5 [2-14]#‡ | 9 [2-60]#‡ |
|  |  | Upper-middle income | 46 [14-60]$‡ | 37 [12-60]*‡ | 33 [11-60]*‡ | 60 [16-60]#* | 60 [21-60]#* |  | 13 [7-44]‡ | 12 [7-26]‡ | 12 [7-30]‡ | 14 [7-60]#‡ | 17 [7-60]#‡ |
|  |  | High income | 36 [14-60]$ | 24 [12-60]* | 29 [14-60]# | 45 [14-60]#* | 60 [22-60]#* |  | 12 [6-30] | 10 [6-23] | 11 [6-27]# | 12 [7-30]# | 27 [8-60]# |

Significant at 5% level: # vs. No AKI in the same group (sepsis or no sepsis), * vs. No sepsis patients with the same RIFLE classification, ‡ vs. West Europe or High income with the same RIFLE classification or the total population, in the same group (sepsis or no sepsis), $ vs. all patients without sepsis.

**Table S6. Mortality per renal replacement therapy modality**

|  | **Total population** | **Sepsis** | **No sepsis** |
| --- | --- | --- | --- |
| ICU mortality, n (%) |  |  |  |
| Haemodialysis | 95 (25.6) | 49 (34.5)*‡ | 46 (20.1)#‡ |
| Haemofiltration | 124 (38.9) | 78 (45.6)* | 46 (31.1) |
| Mixed | 105 (33.4) | 66 (34.0) | 39 (32.5) |
| Hospital mortality, n (%) |  |  |  |
| Haemodialysis | 114 (32.0) | 58 (42.6)* | 56 (25.5)‡ |
| Haemofiltration | 143 (46.1) | 90 (52.9)* | 53 (37.9) |
| Mixed | 129 (42.2) | 88 (47.1)* | 41 (34.5) |

Significant at 5% level: * vs. No sepsis patients, ^#^ vs. Mixed, ‡ vs. Haemofiltration in the same group.

ICU – intensive care unit; n – number of patients

**Table S7. Baseline demographic characteristics of patients with chronic renal failure, with and without sepsis**

|  | **All patients** | **Sepsis** | **No sepsis** | **P-value** |
| --- | --- | --- | --- | --- |
| Number of patients, (%) | 845 (100) | 263 (31.3) | 582 (63.8) | < 0.001 |
| Age, mean (SD) years | 67.7 (14.3) | 67.9 (13.0) | 67.6 (14.9 | 0.76 |
| Male, n (%) | 518 (61.8) | 152 (58.7) | 366 (63.2) | 0.21 |
| Weight, mean (SD) kg | 76.1 (23.0) | 76.0 (19.8) | 76.1 (24.3) | 0.94 |
| Height, mean (SD) cm | 167.4 (9.9) | 167.2 (10.3) | 167.5 (9.7) | 0.72 |
| Severity scores, mean (SD) |  |  |  |  |
| APACHE II | 25.6 (8.8) | 28.9 (8.0) | 24.1 (8.7) | < 0.001 |
| Non-renal APACHE II | 19.6 (8.2) | 22.1 (7.7) | 18.5 (8.2) | < 0.001 |
| SOFA | 8.0 (4.4) | 9.9 (3.8) | 7.1 (4.4) | < 0.001 |
| Non-renal SOFA | 5.4 (3.9) | 7.0 (3.7) | 4.7 (3.8) | < 0.001 |
| Type of admission, n (%) |  |  |  | 0.19 |
| Surgical (non-trauma) | 261 (32.6) | 74 (28.8) | 187 (34.4) |  |
| Medical | 523 (65.4) | 180 (70.0) | 343 (63.2) |  |
| Trauma | 9 (1.1) | 1 (0.4) | 8 (1.5) |  |
| Other | 7 (0.9) | 2 (0.8) | 5 (0.9) |  |
| Source of admission, n (%) |  |  |  | 0.002 |
| Other hospital | 73 (8.6) | 29 (11.0) | 44 (7.6) |  |
| ER/ambulance | 268 (31.7) | 83 (31.6) | 185 (31.8) |  |
| OR/Recovery | 138 (16.3) | 33 (12.5) | 105 (18.0) |  |
| Hospital floor | 307 (36.3) | 110 (41.8) | 197 (33.8) |  |
| Other | 59 (7.0) | 8 (3.0) | 51 (8.8) |  |
| Reason for admission, n (%) |  |  |  | 0.001 |
| Neurological | 50 (5.9) | 8 (3.0) | 42 (7.2) |  |
| Respiratory | 148 (17.5) | 63 (24.0) | 85 (14.6) |  |
| Cardiovascular | 346 (40.9) | 117 (44.5) | 229 (39.3) |  |
| Renal/Ob-gyn | 97 (11.5) | 24 (9.1) | 73 (12.5) |  |
| Haematological | 7 (0.8) | 1 (0.4) | 6 (1.0) |  |
| Digestive/Liver | 107 (12.7) | 33 (12.5) | 74 (12.7) |  |
| Metabolic | 30 (3.6) | 6 (2.3) | 24 (4.1) |  |
| Trauma | 19 (2.2) | 2 (0.8) | 17 (2.9) |  |
| Other diseases | 41 (4.9) | 9 (3.4) | 30 (5.2) |  |
| Comorbidities, n (%) |  |  |  |  |
| COPD | 127 (15.0) | 40 (15.2) | 87 (14.9) | 0.92 |
| Cancer | 88 (10.4) | 25 (9.5) | 63 (10.8) | 0.56 |
| Metastatic cancer | 18 (2.1) | 6 (2.3) | 12 (2.1) | 0.84 |
| Haematologic cancer | 20 (2.4) | 9 (3.4) | 11 (1.9) | 0.18 |
| Insulin-DM | 210 (24.9) | 74 (28.1) | 136 (23.4) | 0.14 |
| Heart failure, NYHA III-IV | 201 (23.8) | 65 (24.7) | 136 (23.4) | 0.67 |
| HIV infection | 8 (0.9) | 4 (1.5) | 4 (0.7) | 0.25 |
| Cirrhosis | 37 (4.4) | 11 (4.2) | 26 (4.5) | 0.85 |
| Immunosuppression | 66 (7.8) | 21 (8.0) | 45 (7.7) | 0.9 |
| Steroid therapy | 58 (6.9) | 15 (5.7) | 43 (7.4) | 0.37 |
| Chemotherapy | 18 (2.1) | 4 (1.5) | 14 (2.4) | 0.41 |

Significant at 5% level. SD – standard deviation; n- number of patients; SOFA – Sequential Organ Failure Assessment; ER – emergency room; OR – operating room; Ob-gyn – Obstetric-gynaecology; COPD – Chronic Obstructive Pulmonary Disease; DM – Diabetes mellitus; NYHA – New York Heart Association; HIV - human immunodeficiency virus

**Table S8. Baseline clinical characteristics of patients with chronic renal failure, with and without sepsis**

|  | **All patients** | **Sepsis** | **No sepsis** | **P-value** |
| --- | --- | --- | --- | --- |
| Systemic inflammatory response syndrome |  |  |  |  |
| Temperature, n (%) |  |  |  | 0.03 |
| <36 | 52 (6.5) | 17 (6.6) | 35 (6.5) |  |
| 36-38 | 643 (80.8) | 195 (76.2) | 448 (83.0) |  |
| >38 | 101 (12.7) | 44 (17.2) | 57 (10.6) |  |
| Leucocytes (x10^9^/L), n (%) |  |  |  | 0.002 |
| <4 | 19 (2.4) | 8 (3.1) | 11 (2.1) |  |
| 4-12 | 319 (40.7) | 82 (32.0) | 237 (44.9) |  |
| >12 | 446 (56.9) | 166 (64.8) | 280 (53.0) |  |
| Heart rate, mean (SD) bpm | 104.6 (23.9) | 109.1 (23.6) | 102.4 (23.8) | < 0.001 |
| Mean arterial pressure, mean (SD) mmHg | 97.1 (21.3) | 95.9 (21.2 | 97.7 (21.3) | 0.26 |
| Respiratory rate, mean (SD) bpm | 25.2 (8.0) | 26.3 (8.0) | 24.6 (8.0) | < 0.01 |
| Patients on ventilator, n (%) | 388 (45.9) | 153 (58.2) | 235 (40.4) | < 0.001 |
| Blood parameters |  |  |  |  |
| FiO2, mean (SD) | 55.3 (26.4) | 58.9 (26.6) | 53.5 (26.2) | < 0.01 |
| PaO2, mean (SD) mmHg | 139.6 (77.3) | 133.7 (72.7) | 142.7 (79.4) | 0.14 |
| PaCO2, mean (SD) mmHg | 41.2 (15.3) | 41.0 (16.8) | 41.3 (14.5) | 0.84 |
| Bicarbonate, mean (SD) mmol/L | 22.2 (5.7) | 21.9 (6.1) | 22.4 (5.5) | 0.31 |
| Arterial pH, mean (SD) | 7.4 (0.1) | 7.4 (0.1) | 7.4 (0.1) | 0.15 |
| Creatinine, mean (SD) mg/dL | 4.2 (3.6) | 4.3 (3.2) | 4.2 (3.8) | 0.79 |
| Potassium, mean (SD) mmol/L | 4.8 (1.0) | 4.7 (0.9) | 4.8 (1.0) | 0.08 |
| Sodium, mean (SD) mmol/L | 139.3 (6.2) | 139.6 (6.7) | 139.2 (5.9) | 0.5 |
| Urea, median [IQR] mmol/L | 37.0 [0.3-90.0] | 41 [0.3-94.5] | 36.0 [0.3 - 87.0] | 0.27 |
| Haematocrit, median [IQR] L/L | 30.9 [26.9-35.9] | 30.0 [26.1-34.7] | 31.0 [27.0 - 36.0] | 0.09 |
| Bilirubin, median [IQR] mg/dL | 0.8 [0.5-1.7] | 0.8 [0.5-1.9] | 0.8 [0.5 - 1.6] | 0.11 |
| Noradrenaline infusion rate, median [IQR] µg/kg/min | 0.0 [0.0-0.1] | 0.0 [0.0-0.2] | 0.0 [0.0 - 0.0] | <0.001 |
| Inotropic score, median [IQR] | 0.0 [0.0-13.0] | 4.0 [0.0-23.0] | 0 [0 - 10] | <0.001 |
| Urine output, median [IQR] mL/24 h | 848 [123-1,718] | 600 [65-1405] | 1000 [150 - 1844] | <0.01 |

Significant at 5% level. Legend: n – number of patients; SD – standard deviation; IQR – interquartile range.

**Table S9. Outcome of patients with chronic renal failure at admission**

|  | **Sepsis**  **(n=263)** | **No sepsis**  **(n=582)** |
| --- | --- | --- |
| ICU LOS, days | 7 [3-60]* | 3 [2-8] |
| Hospital LOS, days | 43 [14-60]* | 16 [7-60] |
| RRT, n (%) | 133 (50.6)* | 185 (31.8) |
| ICU mortality, n (%) | 75 (28.7)* | 103 (18.5) |
| Hospital mortality, n (%) | 107 (42.0)* | 137 (25.8) |

Significant at 5% level: * vs. patients with No sepsis. ICU – intensive care unit;

LOS – length of stay; RRT – renal replacement therapy; n – number of patients
