# Supplementary figures and images for: A worldwide multicentre evaluation of the influence of deterioration or improvement of acute kidney injury on clinical outcome in critically ill patients with and without sepsis at ICU admission: results from The Intensive Care Over Nations audit
Source: Crit Care. 2018 Aug 3;22:188. doi: 10.1186/s13054-018-2112-z (PMC6091052; doi:10.1186/s13054-018-2112-z)

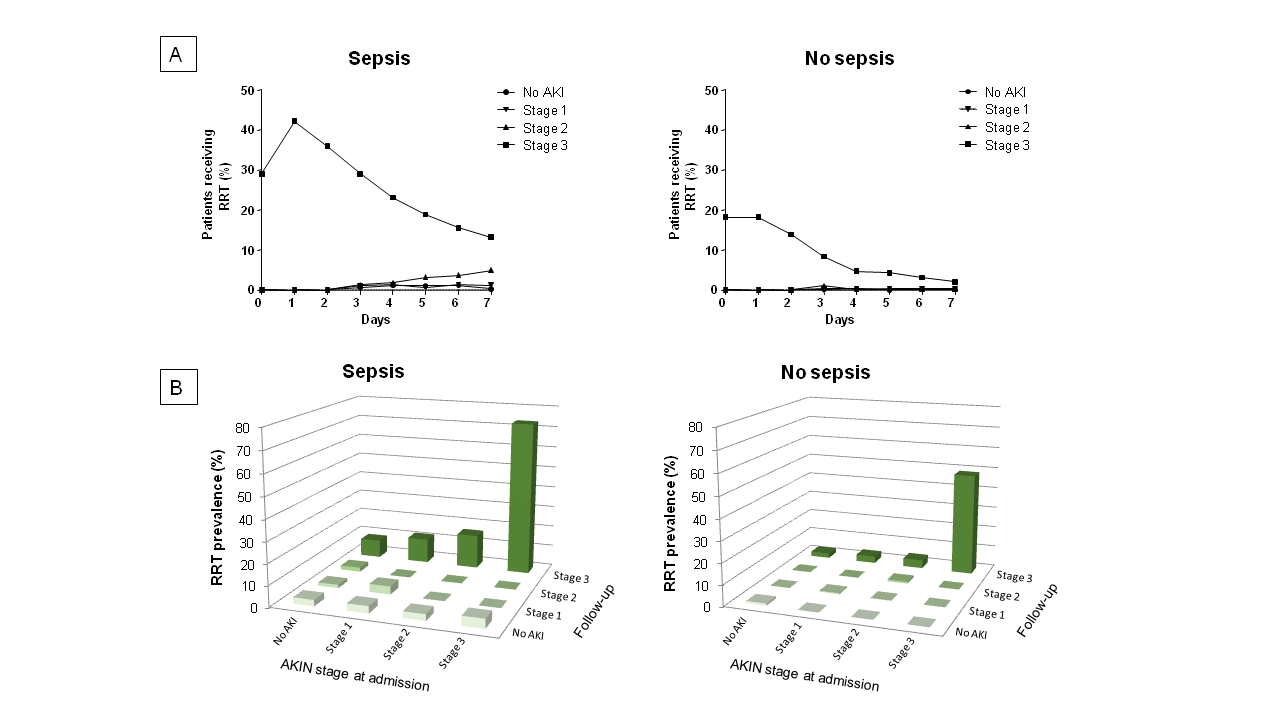

Supplement: Supplementary file 4 — Figure S1. RRT in critically ill patients with and without sepsis. A: Percentage of patients receiving RRT per day per AKIN stage at admission. B: RRT incidence per AKIN stage at follow-up. Admission category presented on x axis. Patients followed-up until day 7 to determine whether they improved to a lower AKIN stage or deteriorated to a worse AKIN stage, presented on z axis. In case of discharge or death, data were imputed by last observation carried forward (PNG 43 kb) [file 13054_2018_2112_MOESM4_ESM.png]

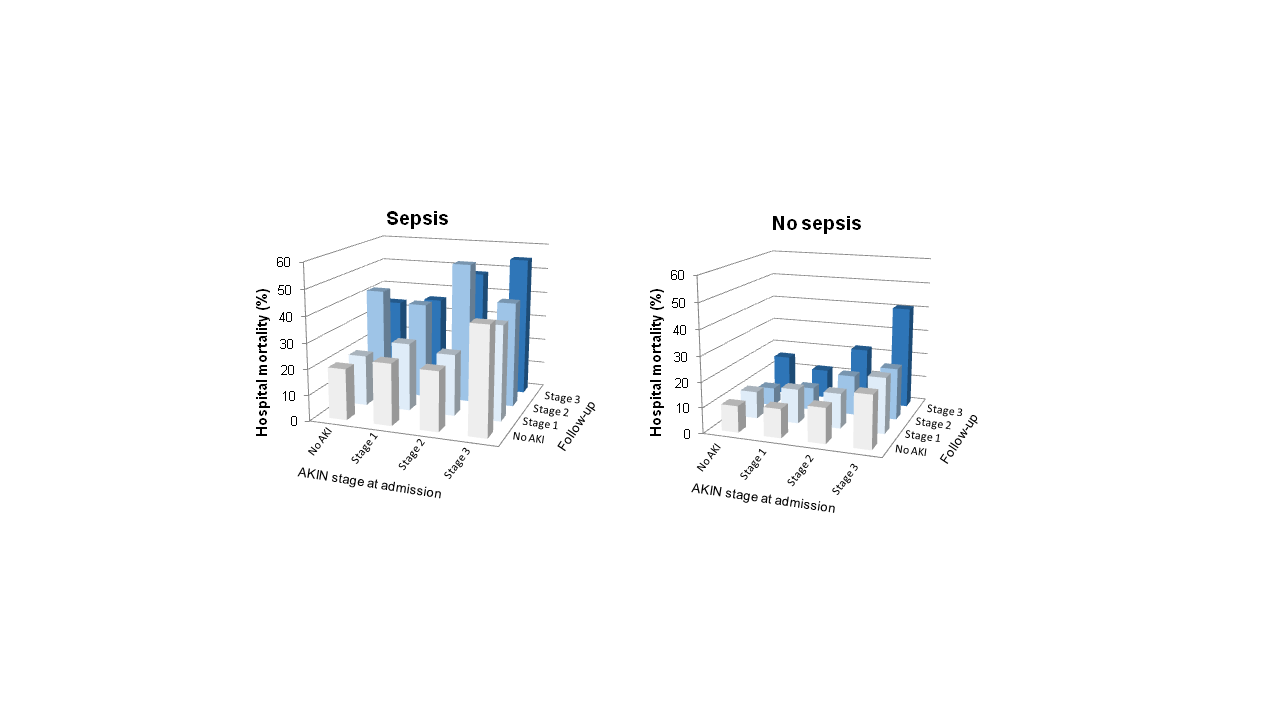

Supplement: Supplementary file 5 — Figure S2. Hospital mortality in septic and non-septic critically ill patients during follow-up. At admission (first 72 hours in ICU), patients were categorized as No AKI and AKIN stages 1, 2 and 3, presented on x axis. Patients followed-up until day 7 to determine whether they improved to a lower AKIN stage or deteriorated to a more severe AKIN stage, presented on z axis. Hospital mortality determined for groups in which patients improved or deteriorated during follow-up. Patients with AKIN stage 3 at admission and at follow-up show highest mortality rate. Worsening of AKI is associated with higher mortality unrelated to AKI category at ICU admission. Graph also shows that patients with AKIN stage 3 at admission who improve to No AKI at follow-up still have twice as high mortality rates compared to patients with No AKI at admission and follow-up (PNG 30 kb) [file 13054_2018_2112_MOESM5_ESM.png]
